# Molecular underpinnings of ssDNA specificity by Rep HUH-endonucleases and implications for HUH-tag multiplexing and engineering

**DOI:** 10.1093/nar/gkaa1248

**Published:** 2021-01-07

**Authors:** Kassidy J Tompkins, Mo Houtti, Lauren A Litzau, Eric J Aird, Blake A Everett, Andrew T Nelson, Leland Pornschloegl, Lidia K Limón-Swanson, Robert L Evans, Karen Evans, Ke Shi, Hideki Aihara, Wendy R Gordon

**Affiliations:** Department of Biochemistry, Molecular Biology, and Biophysics, University of Minnesota Twin Cities, Minneapolis, MN 55455, USA; Department of Computer Science and Engineering, University of Minnesota Twin Cities, Minneapolis, MN 55455, USA; Department of Biochemistry, Molecular Biology, and Biophysics, University of Minnesota Twin Cities, Minneapolis, MN 55455, USA; Department of Biochemistry, Molecular Biology, and Biophysics, University of Minnesota Twin Cities, Minneapolis, MN 55455, USA; Department of Biochemistry, Molecular Biology, and Biophysics, University of Minnesota Twin Cities, Minneapolis, MN 55455, USA; Department of Biochemistry, Molecular Biology, and Biophysics, University of Minnesota Twin Cities, Minneapolis, MN 55455, USA; Department of Biochemistry, Molecular Biology, and Biophysics, University of Minnesota Twin Cities, Minneapolis, MN 55455, USA; Department of Biochemistry, Molecular Biology, and Biophysics, University of Minnesota Twin Cities, Minneapolis, MN 55455, USA; Department of Biochemistry, Molecular Biology, and Biophysics, University of Minnesota Twin Cities, Minneapolis, MN 55455, USA; Department of Biochemistry, Molecular Biology, and Biophysics, University of Minnesota Twin Cities, Minneapolis, MN 55455, USA; Department of Biochemistry, Molecular Biology, and Biophysics, University of Minnesota Twin Cities, Minneapolis, MN 55455, USA; Department of Biochemistry, Molecular Biology, and Biophysics, University of Minnesota Twin Cities, Minneapolis, MN 55455, USA; Department of Biochemistry, Molecular Biology, and Biophysics, University of Minnesota Twin Cities, Minneapolis, MN 55455, USA

## Abstract

Replication initiator proteins (Reps) from the HUH-endonuclease superfamily process specific single-stranded DNA (ssDNA) sequences to initiate rolling circle/hairpin replication in viruses, such as crop ravaging geminiviruses and human disease causing parvoviruses. In biotechnology contexts, Reps are the basis for HUH-tag bioconjugation and a critical adeno-associated virus genome integration tool. We solved the first co-crystal structures of Reps complexed to ssDNA, revealing a key motif for conferring sequence specificity and for anchoring a bent DNA architecture. In combination, we developed a deep sequencing cleavage assay, termed HUH-seq, to interrogate subtleties in Rep specificity and demonstrate how differences can be exploited for multiplexed HUH-tagging. Together, our insights allowed engineering of only four amino acids in a Rep chimera to predictably alter sequence specificity. These results have important implications for modulating viral infections, developing Rep-based genomic integration tools, and enabling massively parallel HUH-tag barcoding and bioconjugation applications.

## INTRODUCTION

HUH-endonucleases, so named for a conserved histidine–hydrophobic residue–histidine (HUH) motif, are diverse enzymes utilizing common single-stranded DNA (ssDNA) processing mechanisms that break and join DNA to facilitate fundamental biological processes such as rolling circle replication (RCR), rolling hairpin replication (RHR), bacterial conjugation, DNA transposition, and DNA integration into host genomes (1–3). At the heart of DNA processing of all HUH-endonucleases is a structurally defined catalytic nickase domain that first recognizes a specific sequence/structure of DNA; nicks ssDNA at a ‘*nic* site’ to yield a sequestered 5′ end that remains covalently bound to the HUH endonuclease and a free 3′OH that can be used as a primer for DNA replication; and finally, facilitates a strand transfer reaction to resolve the covalent intermediate (1). The two major classes of HUH-endonucleases are replication initiator proteins (Reps) involved in RCR and RHR and relaxases involved in bacterial conjugation of plasmids, although HUH-endonucleases are also involved in DNA transposition (1).

The covalent phosphotyrosine intermediate has recently been exploited for biotechnology applications. ‘HUH-tag’ fusion proteins are emerging as a versatile bioconjugation platform to covalently link proteins to DNA, combining the diverse functionality of proteins with the programmability of DNA (4). HUH-tag applications have permeated into technologies such as DNA origami scaffolded protein assembly (5–8), receptor-specific cell targeting by adeno-associated virus (9), aptamer-based sandwich detection (10), directed nanoparticle drug-delivery via DNA aptamers (11), and CRISPR–Cas9 genome engineering (12,13), mainly due to their ability to form robust covalent adducts under physiologic conditions. Rather than relying on expensive nucleic acid modifications such as the SNAP-tag (14), CLIP-tag (15) and HALO-tag (16) systems, HUH-tags rely on an inherent ssDNA binding moiety that promotes the catalysis of a transesterification reaction resulting in a stable phosphotyrosine adduct (1).

Understanding the molecular basis of DNA recognition by HUH-endonucleases could provide much needed solutions for bacterial antibiotic resistance resulting from HUH-endonuclease mediated horizontal gene transfer (17), as well as in the prevention or treatment of HUH-endonuclease mediated viral infections, such as geminivirus infections of plants that ravage the agricultural crop industry (18,19) and parvovirus B19 infections of humans (20) that are associated with a range of autoimmune diseases (21,22). Moreover, the ability to rationally engineer HUH-endonucleases to recognize a desired DNA sequence has huge potential in genome engineering (23) and DNA delivery applications as well as in expanding the multiplexibility of HUH-tagging to meet the demand of the recent explosion of DNA-barcoding applications (24–27).

However, while several structures of relaxase HUH-endonucleases in complex with their cognate DNA target sequences have been reported (17,28–30), there are no structures of viral Rep HUH-endonucleases in complex with ssDNA comprising the target sequence at the origin of replication (*ori*). Despite structurally superimposable active sites and a common overall core structure (31), there are several structural elements of the larger relaxase proteins that do not exist in Reps, such as extensions of the C-terminus and internal loops with respect to Reps. These structures form extensive contacts with the target DNA, thus underscoring potential differences in DNA recognition mechanisms between Reps and relaxases (32).

In this study, we determined the structural basis for ssDNA recognition by viral Rep HUH-endonucleases by solving two Rep-ssDNA co-crystal structures and identified a ssDNA ‘bridging’ motif largely responsible for DNA recognition. This bridging motif recognizes specific bases of bent ssDNA located on either side of the *nic* site using surface pockets. To further interrogate the ssDNA specificity of Reps, we developed HUH-seq, a high-throughput, next generation sequencing (NGS)-based DNA cleavage assay that we used to define ssDNA recognition profiles of a panel of ten Reps using a ssDNA library containing 16,384 different target sequences. Despite the high similarity of cognate nonanucleotide *ori* sequences and the promiscuous nature of Rep ssDNA recognition we noticed previously (4) and further defined in this study, HUH-seq analysis surprisingly revealed many examples of orthogonal adduct formation between Reps from different viral families with little or no cross-reactivity. Finally, we rationally engineered a chimeric Rep by swapping a few amino acids of the ssDNA ‘bridging’ motif of one Rep into the backbone of a related Rep, predictably modulating ssDNA sequence specificity.

## MATERIALS AND METHODS

### Molecular cloning, protein expression and purification

The N-terminal nickase domain of all Reps (Supplementary Table S1) were synthesized as *E. coli* codon-optimized gene blocks from Integrated DNA Technologies (IDT) and designed with 15 nucleotides on each end that were homologous to regions of the linearized pTD68/His6-SUMO parent vector digested with BamHI and XhoI. Final His6-SUMO-Rep constructs were created with the In-Fusion HD Cloning Kit (Takara) and sequence confirmed with Sanger sequencing (Genewiz). Purified plasmids were transformed into BL21(DE3) *E. coli* competent cells (Agilent), initially cultured in 1 l LB broth at 37°C, then induced at OD_600_ with 0.5 mM IPTG (isopropyl-d-1-thio-galactopyranoside, Sigma Aldrich), and then grown for 16 hours at 18°C. Collected cell pellets were resuspended in 10 ml of lysis buffer (50 mM Tris pH 7.5, 250 mM NaCl, 1 mM EDTA, cOmplete protease inhibitor tablet (Pierce) and pulse sonicated for several one minute rounds. The suspension was centrifuged at 24 000 × g for 25 min, and supernatants were batch bound for 1 h with 2 ml HisPure Ni-NTA agarose beads (ThermoFisher) and equilibrated with wash buffer (50 mM Tris pH 7.5, 250 mM NaCl, 1 mM EDTA, 30 mM imidazole). After lysate cleared the gravity column, beads were washed with 30 ml wash buffer, and proteins were eluted from gravity columns with elution buffer (50 mM Tris pH 7.5, 150 mM NaCl, 1 mM EDTA, 250 mM imidazole). Protein was further purified and buffer exchanged into 50 mM Tris pH 7.5, 150 mM NaCl, 1 mM EDTA using the ENrich SEC70 (Bio-Rad) size exclusion column. Aliquots were stored at −20°C and −80°C at 30 μM. SUMO-cleaved recombinant PCV2^Y96F^ and WDV^Y106F^ stocks for crystal screening were prepared in a similar manner as above, however Ni-NTA fractions were dialyzed into 50 mM Tris pH 7.5, 300 mM NaCl, 1 mM EDTA with the addition of 1 mM DTT and SUMO-cleaving protease ULP-1 at 5 U per 1 l of *E. coli* overnight at 4°C. Dialyzed samples were batch bound a second time with Ni-NTA beads and were flowed through a gravity column to remove cleaved His6-SUMO and His6-ULP-1. Protein was concentrated with spin concentrators (Amicon Ultra-15 Centrifugal Filter Unit, 3 kDa cut-off) to 16 mg/ml.

### Crystallization, data collection and processing

An 8-mer oligonucleotide (5′-dAATATTAC-3′) from part of the geminivirus origin of replication sequence was reconstituted in ddH_2_O at 10 mM and mixed with recombinant WDV^Y106F^. We used Rigaku's CrystalMation system to perform a broad, oil-immersion, sitting drop screen of the protein–DNA mixture in the presence of either magnesium or manganese. Crystals were achieved using 8 mg/ml protein solution containing 1.1-fold 8-mer and 5 mM MnCl_2_ with a well solution of 12% (w/v) PEG 8000 precipitating agent, 0.2 mM zinc acetate, and 0.1 M sodium cacodylate at pH 6.5. The crystals belong to space group *P*4_1_2_1_2 with unit cell dimensions of *a* = *b* = 50.63 Å, *c* = 241.98 Å. Addition of any cryoprotectant to these crystals resulted in poor diffraction; the crystals seemed to collapse upon vitrification. Our solution to this issue was to collect datasets using an in-house, X-ray diffractometer (Rigaku Micromax-007 Rotating Anode, Rigaku Saturn 944 CCD Detector) at room temperature. Radiation caused minimal crystal damage, and over 100 frames could be obtained from a single crystal. All data was processed with the HKL suite.

WDV^Y106F^ + 10-mer crystals were also obtained with 1:1 protein solution to well solution, where the well solution was constant (12% (w/v) PEG 8000 precipitating agent, 0.2 mM zinc acetate, and 0.1 M sodium cacodylate at pH 6.5), containing 1mM 10-mer oligonucleotide (5′-dTAATATTACC-3′). Protein and MnCl_2_ concentration, 8 mg/ml and 5 mM respectively, were also held constant. Crystals were soaked in 25% glycerol, and a dataset was collected at the APS Beamline 24 (NE-CAT). Crystals diffracted to 1.8 Å and belong to the *P*2_1_2_1_2_1_ space group with unit-cell parameters: *a* = 45.57 Å, *b* = 50.01 Å, *c* = 73.44 Å. One complex was present per asymmetric unit.

We also used Rigaku's CrystalMation system's broad, sitting drop screen to identify potential conditions for PCV^Y96F^ + 10-mer crystallization. The protein solution contained 8 mg/ml protein, 1 mM 10-mer oligonucleotide (5′-dTAGTATTACC-3′), and 5 mM MnCl_2_. Small needle crystals were obtained with 1:1 protein solution in a well solution of 0.1 M ammonium acetate; 25% polyethylene glycol 3,350; 0.1 M Bis–Tris pH 7. Crystals were soaked in 25% glycerol, and a dataset was collected at the APS Beamline 24 (NE-CAT). Crystals diffracted to 2.03 Å and belong to the *P*6_4_ space group with unit-cell parameters: *a* = *b* = 99.53 Å, *c* = 73.70 Å. There were three complexes per asymmetric unit.

### Structure solution and refinement

The WDV^Y106F^ + 8-mer structure was solved with the molecular replacement function in PHENIX using our previously solved structure of apo WDV Rep (PDB ID: 6Q1M) as a model. We visualized the electron density map using Coot (33) and modeled the 8-mer into a clear electron density tunnel (Supplementary Figure S1A). All eight nucleotides of the oligonucleotide were unambiguously built into well-defined electron density of each of the two complexes in the asymmetric unit. Subsequent refinement was performed with default settings of PHENIX auto.refine with NCS applied (34) and alternated with visual inspection and model correction. Final R-work and R-free were 0.188 and 0.246, respectively.

The WDV^Y106F^ + 10-mer, *P*2_1_2_1_2_1_ structure was solved with the Phaser molecular replacement function in PHENIX using the previously solved WDV^Y106F^ + 8-mer structure. The two additional nucleotides were modeled into appropriate density. Again, Coot was used for model building, and PHENIX auto.refine was used for refinement. The final R-work and R-free were 0.173 and 0.224 respectively.

For the PCV^Y96F^ + 10-mer structure, a model for molecular replacement was generated in PyMol by superimposing the WDV^Y106F^ + 8-mer structure with the Porcine circovirus 2 Rep domain (PDB ID: 5XOR) structure. The 8-mer from the WDV model was added to the PCV Rep domain model and used for Phaser molecular replacement in Phenix. The two additional nucleotides were modeled, and the oligonucleotide sequence was corrected using Coot. PHENIX auto.refine was used for refinement. Two of the complexes in the asymmetric unit had well-defined electron density; density corresponding to the third complex was poorly defined due to inherent crystal properties as demonstrated by comparing simulated annealing omit maps of the active sites from each complex copy (Supplementary Figure S1B–D). As a result, R-values are higher than normal for this resolution structure. R-work and R-free were calculated to 0.229 and 0.280, respectively. An additional 4 nucleotides from a second ssDNA strand was modeled near the surface of the third complex, which seems to be non-specifically bound (Supplementary Figure S1E).

### *In vitro* HUH cleavage assay

Cleavage of the synthetic oligos was carried out using final concentrations of 3 μM SUMO-Rep and between 4.5 and 30 μM oligo in 50 mM HEPES pH 8.0, 50 mM NaCl, and 1 mM MnCl_2_ for 30 min at 37°C. The reactions were quenched with 4x Laemmli buffer containing 5% β-ME, boiled for 5 min at 100°C, and run on a 4–12% SDS-PAGE acrylamide gel. For time course reactions, aliquots were removed from an HUH reaction master mix at specified time intervals and immediately quenched in 4× Laemmli buffer containing 5% β-ME. Percent covalent adduct formation was calculated using Bio-Rad ImageLab software. The background subtraction function of ImageJ was used to process all gel images.

### HUH-seq ssDNA library cleavage, library preparation, and sequencing

A 90-nt ssDNA library with a central 7 base randomized region flanked by conserved regions harboring primer binding sites at either termini (7N ssDNA library) was constructed using IDT oPools service consisting of 128 individually synthesized DNA oligos mixed at equal molarity, producing a ssDNA library containing 16,384 sequences (extended data 1). Recombinant Rep cleavage of the 7N ssDNA library was carried out in triplicate in 3 μM Rep and 300 nM (83.4 ng/μl) ssDNA library in 50 mM HEPES pH 8.0, 50 mM NaCl and 1 mM MnCl_2_ for 1 h at 37°C. The Rep enzymes were immediately heat inactivated by boiling at 95°C for 3 min. The remaining uncleaved ssDNA library from each Rep *in vitro* cleavage reaction was diluted 10-fold in water and amplified using 0.5 μM TruGrade/HPLC purified primers from IDT containing Nextera adapters and spacer regions with 2× CloneAmp™ HiFi PCR Premix for 30 cycles. The resulting product was a 200 bp dsDNA amplicon run on a 1.5% agarose gel and stained with SybrSafe. Each 200 bp product was gel extracted (NucleoSpin Gel and PCR Clean-up kit, Macherey-Nagel) and eluted in 30 μL NE elution buffer (5 mM Tris–HCl, pH 8.5) resulting in samples of 30–60 ng/μl. All samples were barcoded with Illumia dual-indexing sequences via the Nextera adapters (University of Minnesota Genomics Core). Indexed samples are were pooled and run on a 1.5% agarose gel; the 270 bp barcoded pooled sample was gel extracted and then sequenced using a single Illumina HiSeq lane (350 000 000 paired-end reads, Genewiz) spiked with 30% PhiX to prevent molecule clumping to ensure a balanced fluorescent signal. This improves overall run quality due to low library diversity (i.e. every amplicon has the same constant region composition).

### HUH-seq read count reduction analysis and sequence logo generation

Raw NGS sequence data were processed using R. Non-randomized portions (e.g. adapter sequences and constant regions) were removed from each read to extract only the randomized 7-mer (*k*-mer). 7-mers from reverse reads were reverse-complemented, and frequency counts for each of the 16 384 unique 7-mers were generated for the reference library from each of the Rep treatment libraries. Each treatment was then compared against the reference to estimate a log_2_-fold-change and percent reduction (reference – treatment/ reference) for each of its 7-mers (extended data 2). The percent reduction data was used to generate weighted sequence logos for each Rep using the ggseqlogo package in R. In addition, log counts per million (logCPM), one-way ANOVA *F*-test statistics (*F*), *P*-values and False Discovery Rate (FDR) statistics were generated using the edgeR package for each *k*-mer per Rep treatment in triplicate (extended data 3). *P*-value and FDR range are between 0 and 1, where a value <0.05 is considered a significant log_2_FC over reference for the respective *k*-mer.

### Extracting predicted orthogonal Reps and *k*-mer sets

Orthogonality of Reps was determined *in silico* using a custom R script. The script first iterates through each Rep and labels it as strongly reactive, moderately reactive, or nonreactive with each of the *k*-mers; any log_2_FC under –3.0 considered strongly reactive, and any over –0.3 considered nonreactive. Then, the number of strongly-reactive-plus-nonreactive *k*-mers is counted for every possible pairing of Reps. Two Reps, A and B, are labeled as ‘likely orthogonal’ if there exists at least one such *k*-mer in each direction—one where A is strongly reactive and B is nonreactive, and another where A is nonreactive and B is strongly reactive.

## RESULTS

### Rep HUH-endonuclease co-crystal structures

**Figure 1.**
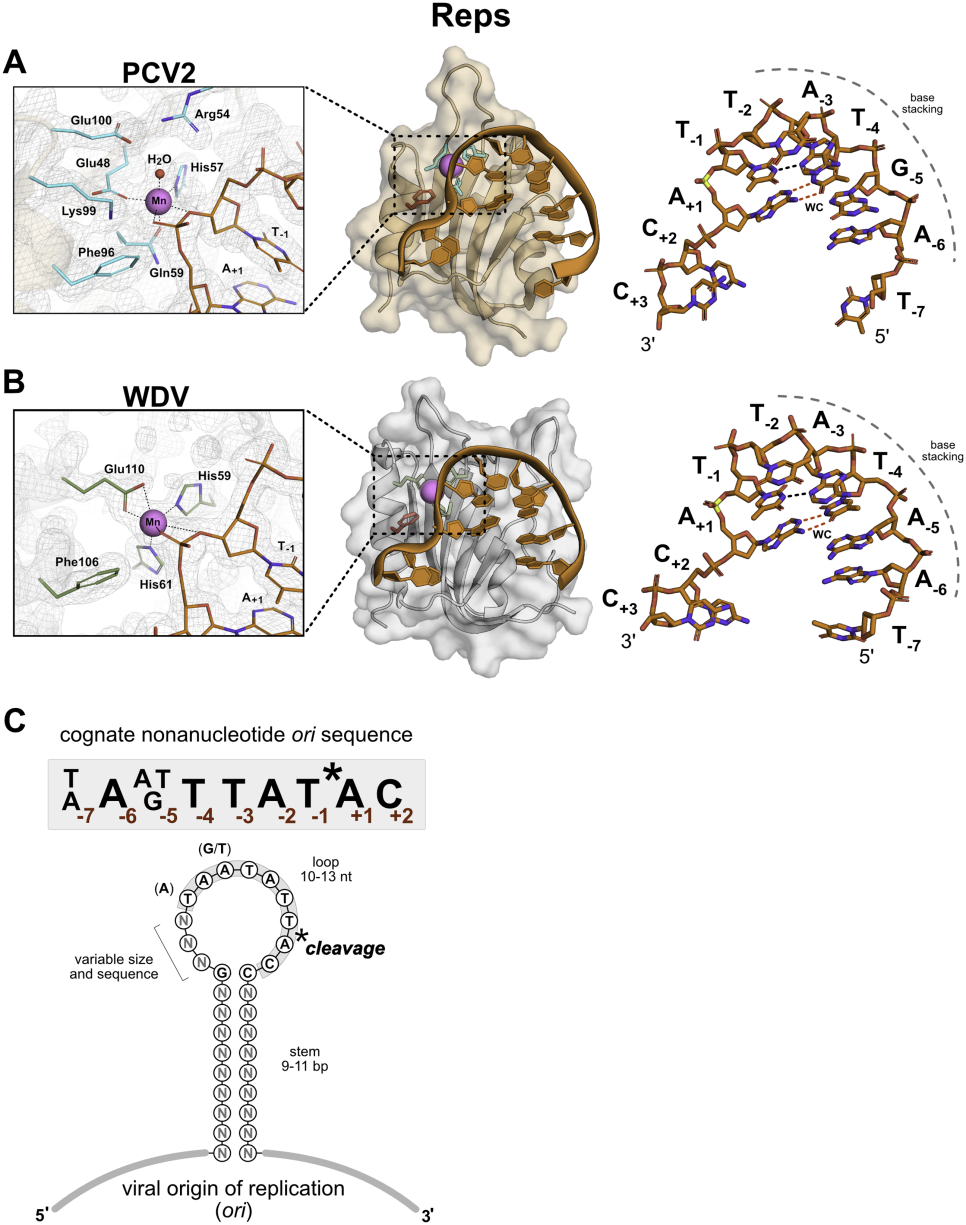
PCV and WDV Rep co-crystal structures complexed with ssDNA target sequence. (**A**) Semi-transparent surface and cartoon representation of PCV2^Y96F^ colored in beige and (**B**), WDV^Y106F^ colored in gray bound to manganese as a sphere in magenta and DNA 10-mers as sticks colored orange by element. PCV2^Y96F^ is bound to 10-mer (5′-dTAGTATTACC-3′), and WDV^Y106F^ is bound to 10-mer (5′-dTAATATTACC-3′) both adopting a U-shaped conformation. Nucleotides are labeled as single letter abbreviations and positions, indicated as subscripts, relative to the scissile phosphate in yellow. A dashed gray curve indicates the base stacking chain that occurs between positions –6 through –2. Intramolecular Watson–Crick (WC) base pairing between A_+1_ and T_−4_ are indicated by red dashed lines as well as a non-canonical hydrogen bond between T_−1_ and A_−3_ are indicated as a black dashed line. Active site side chains are indicated as sticks, PCV2^Y96F^ in cyan and WDV^Y106F^ in green by element. The PCV2^Y96F^ active site coordinates the manganese in an octahedral geometry using Glu48, His57, and Gln59 with a water and two oxygens of the scissile phosphate completing the coordination shown as black dashed lines. The WDV^Y106F^ active site coordinates the manganese in an octahedral geometry using Glu110, His59, and His61 with two oxygens of the scissile phosphate completing the coordination shown as black dashed lines. The active site is displayed within the 2mFo-DFc map mesh at σ = 2. (**C**) Consensus cognate nonanucleotide *ori* sequence of 10 different Reps from *Circoviridae, Nanoviridae and Geminiviridae* (Supplementary Table S1). The origin of replication (*ori*) from these ssDNA viruses contains a stem-loop hairpin with Rep cleavage occurring between position –1 and +1 within the nonanucleotide sequence. The viral *ori* contains a stem that varies in sequence and between 9–11 base pairs in length while the loop contains the cognate nonanucleotide sequence and varies between 10 and 13 nucleotides in length.

To uncover the ssDNA recognition mechanism of Reps and identify potential motifs that might confer sequence specificity, we solved the first high resolution crystal structures of two Rep nickase domains from Porcine circovirus 2 (PCV2) and Wheat dwarf virus (WDV) with 12% sequence identity, bound to ssDNA encoding minimal sequences comprising the respective origin of replication (*ori*) sequences. The pre-cleavage state was captured by mutating the catalytic tyrosine to phenylalanine. We present structures of two 10-mer bound structures of inactive PCV2^Y96F^ (1.93 Å resolution) and WDV^Y106F^ (1.80 Å resolution), and one 8-mer bound WDV^Y106F^ (2.61 Å resolution) structure (Table 1). All three structures are in complex with the divalent cofactor manganese. Additionally, the catalytic tyrosine (though a Phe mutant in the structures) is positioned for nucleophilic attack of the scissile phosphate, where the active oxygen of PCV2 and WDV is measured at 2.2 and 2.9 Å from the phosphate, respectively (Figure 1A and B, Supplementary Note S1).

**Table 1.** Data collection and refinement statistics

	PCV^Y96F^ + 10mer^a^ (PDB 6WDZ)	WDV^Y106F^ + 10mer^a^ (PDB 6WE0)	WDV^Y106F^ + 8mer^a^ (PDB 6WE1)
***Data collection***			
Wavelength (Å)	0.979	0.979	1.542
Resolution range (Å)	43.1–2.03 (2.10–2.03)^b^	41.34–1.8 (1.86–1.8)^b^	28.55–2.61 (2.71–2.61)^b^
Space group	*P*6_4_	*P*2_1_2_1_2_1_	*P*4_1_2_1_2
Unit cell (Å)	*a* = *b* = 99.53, *c* = 73.70, 90°, 90°, 120°	*a* = 45.57, *b* = 50.01, *c* = 73.44, 90°, 90°, 90°	*a* = *b* = 50.63, *c* = 241.98, 90°, 90°, 90°
Unique reflections	26261 (2625)	15366 (6674)	9674 (876)
Completeness (%)	97.52 (98.17)	95.23 (98.54)	93.29 (87.60)
Wilson *B*-factor	25.87	26.51	53.18
Mean I/σ	12.49 (3.63)	14.17 (2.05)	7.70 (2.27)
CC1/2	0.996 (0.812)	0.997 (0.571)	0.987 (0.702)
CC*	0.999 (0.947)	0.999 (0.862)	0.997 (0.908)
*R*-meas	0.0821 (0.441)	0.0836 (0.963)	0.112 (0.609)
*R*-pim	0.0470 (0.248)	0.037 (0.436)	0.071 (0.378)
***Refinement***			
Reflections used in refinement	26 261 (2625)	15 364 (1550)	9674 (876)
Reflections used for *R*-free	1228 (91)	818 (92)	486 (40)
*R*-work	0.229 (0.273)	0.173 (0.220)	0.188 (0.301)
*R*-free	0.280 (0.287)	0.224 (0.313)	0.246 (0.354)
Number of non-hydrogen atoms	3331	1169	2106
Macromolecules	3126	1112	2098
ligands	11	4	3
solvent	194	53	5
Protein residues	302	119	229
RMS (bonds) (Å)	0.009	0.006	0.008
RMS (angles) (°)	1.11	1.09	1.06
Ramachandran favored (%)	97.96	98.29	96.31
Ramachandran allowed (%)	2.04	1.71	3.69
Ramachandran outliers (%)	0	0	0
Rotamer outliers (%)	0	0	0.54
Clashscore	10.64	0	6.27
Average *B*-factor	32.57	30.37	47.17
Macromolecules	32.61	29.89	47.20
Ligands	33.51	34.76	42.20
Solvent	31.84	40.15	34.09

^a^Data are from one crystal.

^b^Values in parentheses are for highest resolution shell.

### Rep docking interface conforms ssDNA to ‘U-shaped’ architecture

Reps involved in RCR are known to cleave in the loop of a DNA hairpin harboring the cognate *ori* sequence (Figure 1C). Strikingly, despite the absence of bases that make up the hairpin stem in the short target DNA oligos, the ssDNA is bent into a ‘U-shaped’ architecture like one might expect in the context of the hairpin loop. The U-shaped DNA sits in a shallow channel on the surface of one face of the Rep protein with a distinct topological ‘nose’ that juts out in the center of the U. The bent conformation of the ssDNA in the Rep structures is driven by both intermolecular interactions with the topological ‘nose’ of the protein and by intramolecular Watson–Crick base pairing between T_−4_ and A_+1_ along with adjacent hydrogen bonding between N3 of T_−1_ and N3 of A_−3_ (Figure 1A and B). Moreover, energetically favorable base stacking occurs between 5 nucleotides at positions −6 through −2. These intramolecular, conformation stabilizing interactions, along with protein–nucleotide interactions, promote the proper orientation needed for catalysis of the 5′ phosphate of the position +1 nucleotide.

**Figure 2.**
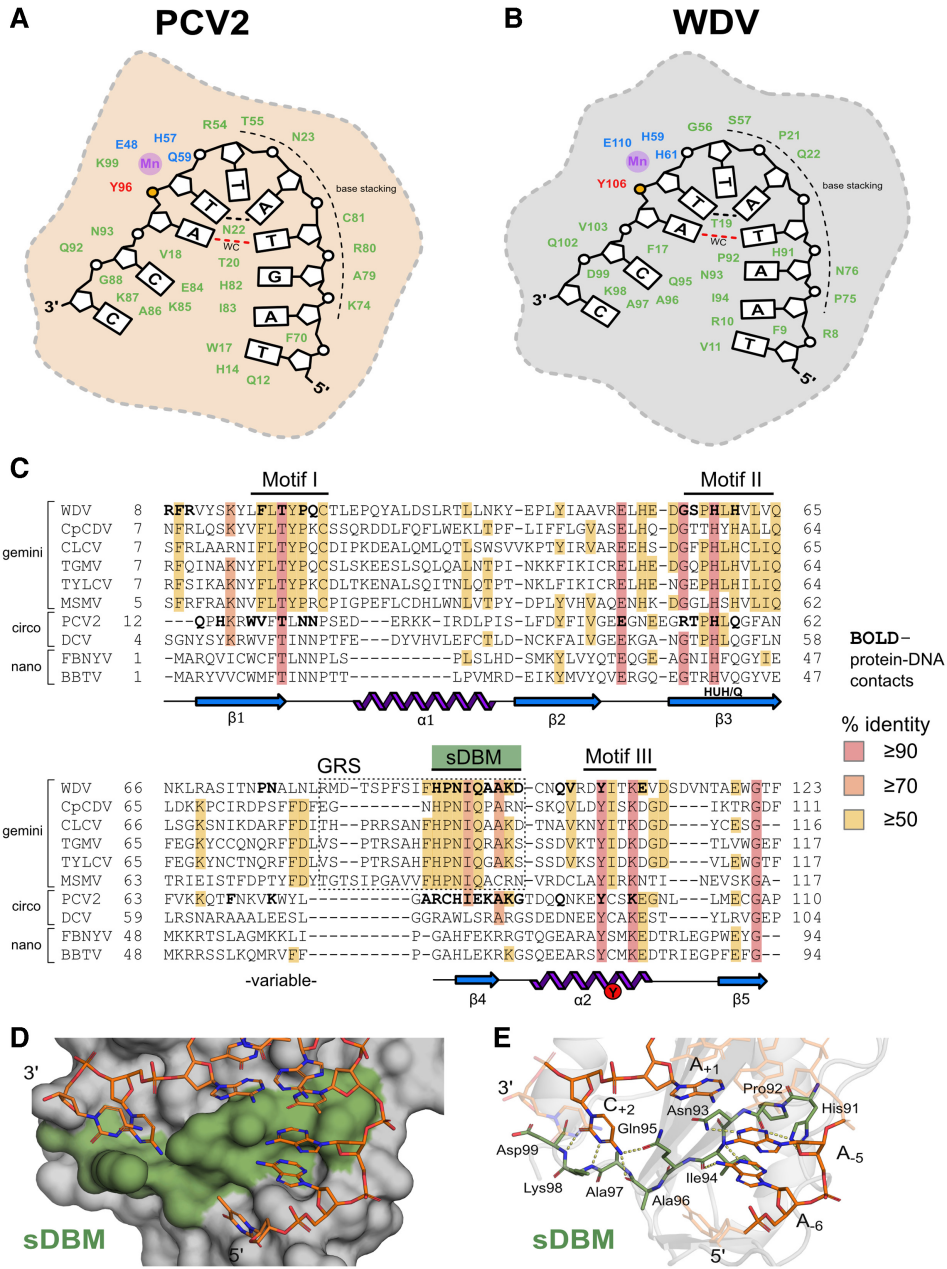
Cartoon depiction and structural alignment of specific Rep protein–DNA interactions: (**A**) PCV2 and (**B**) WDV Rep structures depicted as 2D cartoons with relative positions of residues (green) involved in binding ‘U-shaped’ ssDNA within 4 Å. The catalytic tyrosine 106 is indicated in red with the adjacent phosphate in yellow, the ion coordinating triad is indicated in blue, and the 2+ ion in purple. The single Watson–Crick (WC) base pair is indicated as a dashed red line, and the ssDNA intramolecular hydrogen bond is indicated as a black dashed line. (**C**) Structural alignment of Reps using PROMALS3D including available PCV2 (PDB: 6WDZ), WDV (PDB: 6WE0), TYLCV (1L2M) and FBNYV (6H8O) structures as templates with conserved residues highlighted - high or absolute conservation (≥90%) indicated in red; moderately conserved (≥70%) indicated in orange; low conservation (≥50%) indicated in yellow; and no conservation (<50%) indicated in white. Amino- and carboxy- terminal ends are trimmed to reflect only structured domains in crystal structures. Bolded residues indicate contacts within 4 Å of DNA 10-mers complexed with PCV2^Y96F^ (PDB: 6WDZ) and WDV^Y106F^ (PDB: 6WE0). Conserved Rep Motifs I/II/III are shown as well as the GRS motif within the dashed box for geminivirus Reps. The sDBM we have defined in this study is labelled and highlighted in green. The conserved secondary structural elements making up the core nickase domain (β1–5 and α1–2) below the alignment sequences are shown as 2D cartoons with labeled HUH/Q motif and catalytic tyrosine. (**D**) Surface representation of WDV with sDBM highlighted in green bound to the 10-mer as sticks. (**E**) Major polar interactions between WDV sDBM residues (green sticks) and bases of 10-mer (orange sticks) are shown as yellow dashes.

To analyze the contacts between protein and DNA facilitating sequence-specific ssDNA recognition, we generated protein-nucleotide interaction maps utilizing the DNAproDB platform (35,36), which reports contacts within 4 Å between protein and ssDNA (Supplementary Figure S2). The relative positions of residues directly involved in forming the ssDNA docking interface, the catalytic tyrosine, and the divalent metal coordinating residues of the 10-mer bound Rep structures are depicted as a cartoon (Figure 2A and B) and mapped onto a structure-based alignment of several Reps (Figure 2C). The structural positioning of residues involved in protein-DNA contacts in the PCV2 and WDV are nearly conserved, while the residue identity is more divergent. A majority of the ssDNA docking interface is created by a stretch of 9–10 consecutive residues that partly correspond to the topological ‘nose’ sticking up in the middle of the U, comprising an observed turn-β4-turn structural motif, which resides within a previously defined region termed the geminivirus recognition sequence (GRS) (Figure 2C) (37). A second prominent cluster of protein–DNA contacts reside within Motif I, both of which were previously implicated in DNA binding (37–39).

### Defining the single-stranded DNA bridging motif (sDBM)

The consecutive stretch of 9–10 residues in the turn-*β*4-turn structural motif (‘ARCHIEKAKG’ for PCV2 and ‘HPNIQAAKD’ for WDV) has two critical functions in the structure. First, it acts as a ‘bridge’ between 5′ and 3′ ends of the nonanucleotide sequence contacting positions −6, −5, +1 and +2. (Figure 2D and E). In combination with the intramolecular base pairing and hydrogen bonding of the ssDNA, this sequence of residues likely contributes to bending and stabilizing the ssDNA in the U-shaped conformation. In the WDV^Y106F^ + 10-mer structure, residues His91 and Asp93 in this ‘bridging’ motif specifically contact the base of A_−5_ (Figure 2D and E), whereas Arg79 and His81 in the PCV2 structure contact the base of G_−5_. We hypothesize that these contacts play a major role in conferring specificity differences at the −5 position. Previously, this motif remained undefined across Rep classes because of divergence in sequence conservation, though this divergence may be a major impetus for ssDNA recognition. Further, this motif is located in the N-terminus in relaxases and near the C-terminus of transposases and is involved in ssDNA binding (Supplementary Figure S3). With this, we term this turn-β-turn structural motif as the ‘single-stranded DNA Bridging Motif’ (sDBM), and suggest that it is the main binding moiety responsible for recognition and conformation priming of ssDNA by Rep HUH-endonucleases.

### Rep versus relaxase ssDNA interfaces reveal a reminiscent yet distinct recognition mechanism

**Figure 3.**
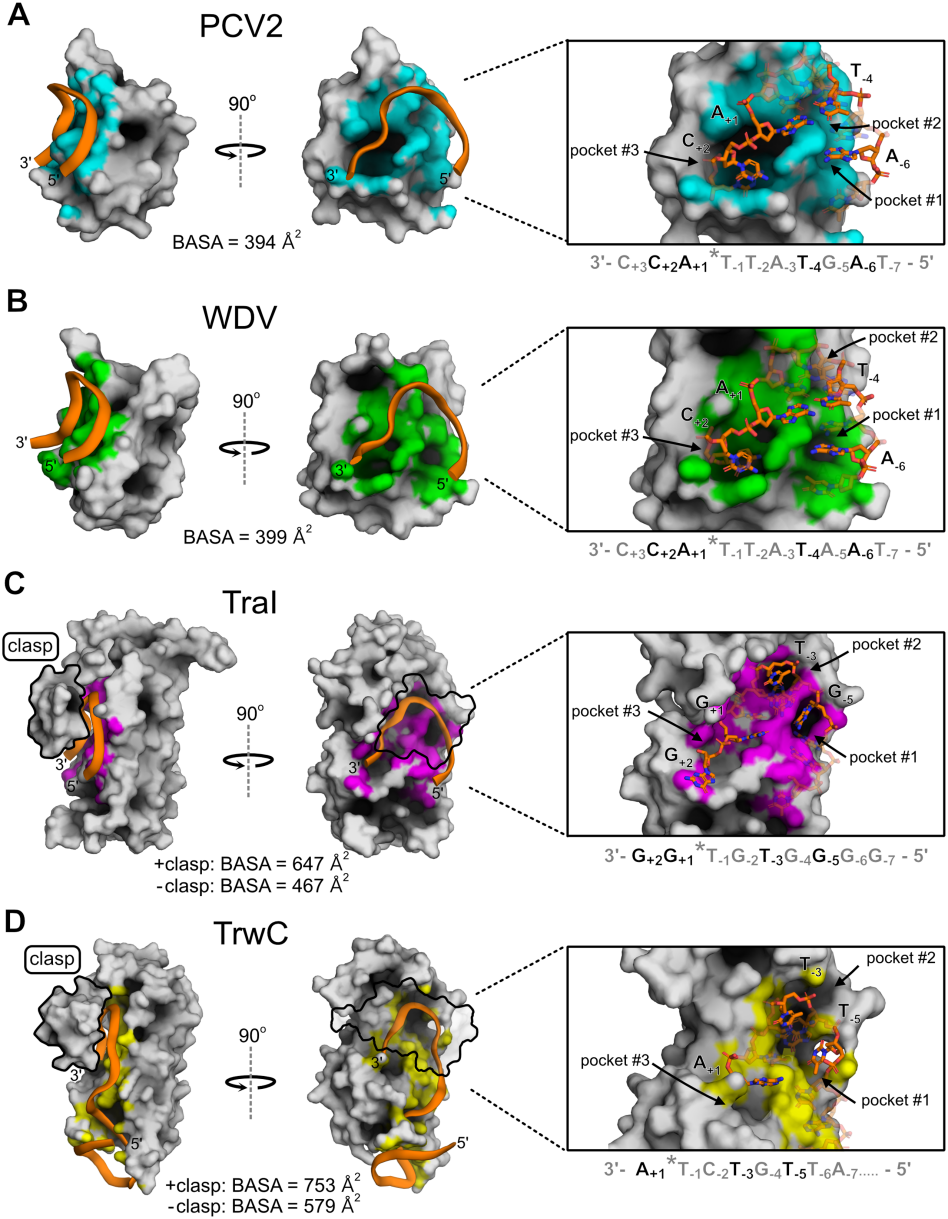
Structural comparison of ssDNA recognition by Reps and relaxases: (**A–D**), PCV2 (6WDZ), WDV (6WE0), TraI36 (2A0I), TrwC (2CDM), are illustrated in gray surface display where DNA interactions within 4 Å are highlighted in cyan, green, magenta, and yellow, respectively. Bound single-stranded DNA is represented as a cartoon backbone in orange or as sticks with carbons and phosphates colored in orange. Nucleotides bound inside pockets are solid; other bound nucleotides are transparent. Relaxase ‘clasps’ (TraI36 residues 231–271 and TrwC residues 237–262) are either solid or transparent and outlined in black. Total buried solvent accessible surface area (BASA; Å^2^) for ssDNA bound to the docking interface was calculated for each structure including values for with, or without, contribution from relaxase clasps.

Reps initiate replication of a large number of viruses and plasmids to copy their circular genomes while relaxases catalyze the transfer of one DNA strand of the plasmid genome to the recipient cell during plasmid conjugation (1); thus, relaxases are thought to recognize DNA with more specificity than Reps. Our structures provide insights at the molecular level into different modes of recognition between Reps and relaxases that should illuminate structural nuances of ssDNA recognition. The two available relaxase structures that are the most comparable to the Rep co-crystal structures are TraI (PDB ID: 2A0I) and TrwC (PDB ID: 2CDM), which are both complexed with ssDNA and have at least one nucleotide bound on the 3′ side of the *nic* site (Figure 3C and D). Structurally, Reps and relaxases share a similar central 5-stranded antiparallel beta-sheet core displaying the HUH motif, though the relaxases are circularly permuted with respect to the Reps such that the catalytic tyrosine is near the C-terminus of Reps and the N-terminus of relaxases (31). Relaxases have similar active sites and U-shaped ssDNA architectures to Reps (28,30), however there are striking differences in how the two families of proteins recognize DNA. Aside from the most obvious difference of a larger size and a more extended DNA binding interface that includes binding a hairpin structure 5′ to the *nic* site of relaxase proteins, the most distinctive difference is that the relaxase structures contain a protein alpha-helical ‘clasp’ that covers the bound DNA (Figure 3C and D). This clasp forms extensive contacts with the DNA, suggesting that it helps anchor the DNA to the protein. This is underscored by the fact that in the crystal structure of NES, the relaxase from *Staphylococcus aureus*, which does not contain a ‘clasp’, the 3′ end of the DNA has very few contacts with the protein (17).

Moreover, the DNA in relaxase proteins is embedded in a much deeper channel than in Rep proteins. Indeed, calculations of buried solvent accessible surface area (BASA) between protein and DNA reveal a more substantial buried surface area in the binding of DNA to relaxases even when accounting for the surface area buried by the clasps (Figure 3). Both Rep and relaxase structures have obvious structurally conserved pockets in the ssDNA docking interface in which individual nucleotide bases are bound. In all structures, the sDBM is a major contributor to the formation of these pockets, which is part of β1 in relaxases and β4 in Reps. TraI and TrwC bury nucleotides –5 and –3 in strikingly deep pockets, #1 and #2, respectively (Figure 3). Reps have pockets in this structural region, yet they are much more shallow and only minimally bury nucleotides at −4 and −6 positions. A_−6_ is bound in the deepest of these Rep pockets, yet it is still oriented in a configuration that favors base stacking with neighboring nucleotides rather than a ‘knob-in-pocket’ interaction as seen in both TrwC and TraI structures. Conversely, both Reps have a deep pocket, #3, where the +2 cytosine base is buried. The only relaxase structure that contains the +2 base is TraI, however the base is not bound in the same conserved pocket (Figure 3).

### HUH-seq uncovers subtle differences in Rep ssDNA recognition specificity

Structural analysis of the Rep protein–DNA contact maps point to subtle differences that contribute to recognition of nearly identical nonanucleotide sequences, suggesting that Reps may differentially tolerate substitutions in the target DNA sequence. Thus, we developed a NGS-based cleavage assay approach, HUH-seq, to examine both ssDNA specificity and to explore expansion of the use of Reps in multiplexed HUH-tag applications. As a first step in assessing the ssDNA recognition specificity of Reps, we asked whether viral Rep proteins from different families and genera (Table 2) differentially tolerate mutations in the target nonanucleotide sequence by measuring covalent adduct formation with an *in vitro* HUH cleavage assay (Supplementary Note S2, Supplementary Figure S4A). However, it became immediately evident that a low-throughput assay would insufficiently characterize specificity due to widespread toleration of variable target sequences. A large number of truncations and substitutions within the nonameric sequence resulted in negligible effects on adduct formation in many cases (a full analysis of the small oligo library screen is provided, Supplementary Figure S4B and C). This realization prompted us to devise a high-throughput method that would reveal ssDNA recognition profiles for each Rep.

**Table 2.** Panel of 10 expressed and purified recombinant Reps

Rep	Viral species	Family	Genus	MW (kDa)	Cognate nonanuclotide *ori* sequence
**PCV2**	*Porcine circovirus 2*	*Circoviridae*	Circovirus	13.1	AAGTATT*AC
**DCV**	*Muscovy duck circovirus*			12.4	TATTATT*AC
**BBTV**	*Banana bunch top virus*	*Nanoviridae*	Babuvirus	11.2	
**FBNYV**	*Faba bean necrotic yellows virus*		Nanovirus	11.3	TAGTATT*AC
**WDV**	*Wheat dwarf virus*	*Geminiviridae*	Mastrevirus	15.6	TAATATT*AC
**CpCDV**	*Chickpea chlorotic dwarf virus*			14.5	
**MSMV**	*Maize striate mosaic virus*			13.4	
**TYLCV**	*Tomato yellow leaf curl virus*		Begomovirus	15.5	
**CLCV**	*Cabbage leaf curl virus*			13.3	
**TGMV**	*Tomato golden mosaic virus*			14.4	

**Figure 4.**
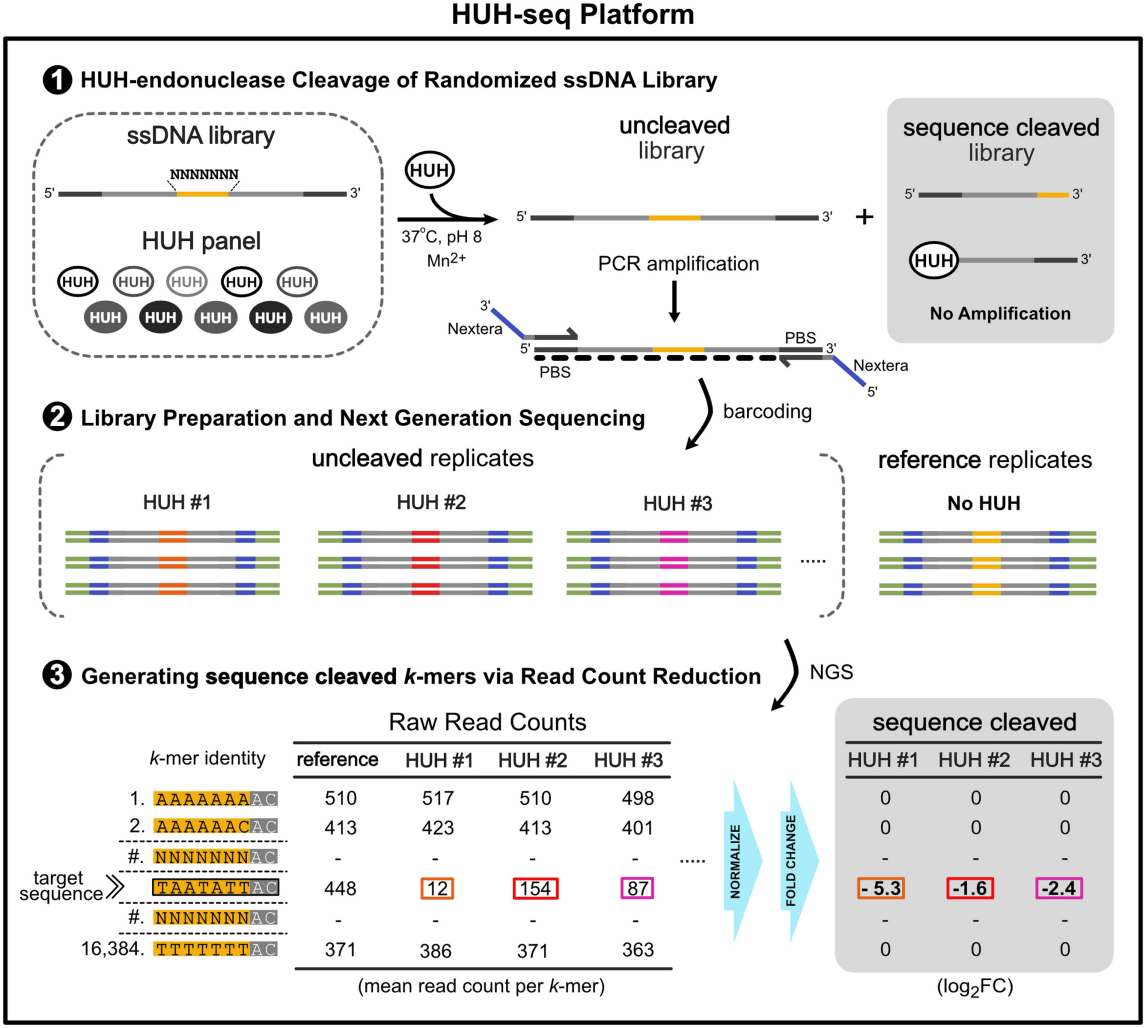
HUH-seq cleavage assay schematic for determining Rep sequence specificity. Schematic describing HUH-seq: an NGS-based approach for quantifying ssDNA specificity profiles of Reps. A synthetic ssDNA library containing seven random bases (4 bases ∧ 7 positions = 16 384 unique kmers) (yellow) flanked by constant regions (gray) and primer binding sites (PBS) (dark gray) are reacted with a panel of Reps, or no enzyme as a reference, in replicate, generating a two part pool containing the ‘uncleaved’ library and the ‘sequence cleaved’ library for each reaction. In a single PCR step, the antisense strand for the ‘uncleaved’ pool is generated, amplified and Nextera adapters (purple) are added with primer overhangs; the ‘sequence cleaved’ library is not amplified due to physical separation of the PBS’s. Each set of amplicons is then barcoded with standard i7/i5 Illumina indexing sequences (green) and pooled for a single next generation sequencing run. A custom R-based analysis script generates read counts for all *k*-mers in each set of replicates, then normalizes based on total read count, and quantifies *k-*mer cleavage extent of each Rep in the panel based on fold change and percent reduction.

To this end, we developed HUH-seq, an NGS-based approach used to establish comprehensive ssDNA recognition profiles of the Reps contained within a randomized ssDNA library containing 16,384 sequences, or *k*-mers. In brief, the first seven positions of the nonanucleotide target sequence are randomized in the 7N ssDNA library, where positions A_+1_ and C_+2_ are constant (‘7N’ - N_−7_N_−6_N_−5_N_−4_N_−3_N_−2_N_−1_*A_+1_C_+2_). The library was constrained to only seven positions in order to limit the size of the library; further design considerations are discussed in the supplementary information (Supplementary Figure S5A, Supplementary Note S2 and Supplementary Equations S1 and S2). Reps were individually reacted with the 7N ssDNA library under standard conditions and produced two populations of the library: ‘sequence cleaved’ and ‘uncleaved’. A primer set containing Nextera adapters was used to generate the antisense strand and to amplify the ‘uncleaved’ population in a single PCR step, while the ‘sequence cleaved’ population remained unamplified. The ‘uncleaved’ amplicons were barcoded with standard dual-indices and sequenced using the HiSeq platform to obtain read counts for every sequence in the library (*k*-mer). Read counts from reference replicates (no Rep added to the reaction) were used to calculate log_2_-fold-change (FC) and read count percent reduction based on the difference between the normalized reference library read counts and normalized ‘uncleaved’ read counts for each Rep treatment (Figure 4).

**Figure 5.**
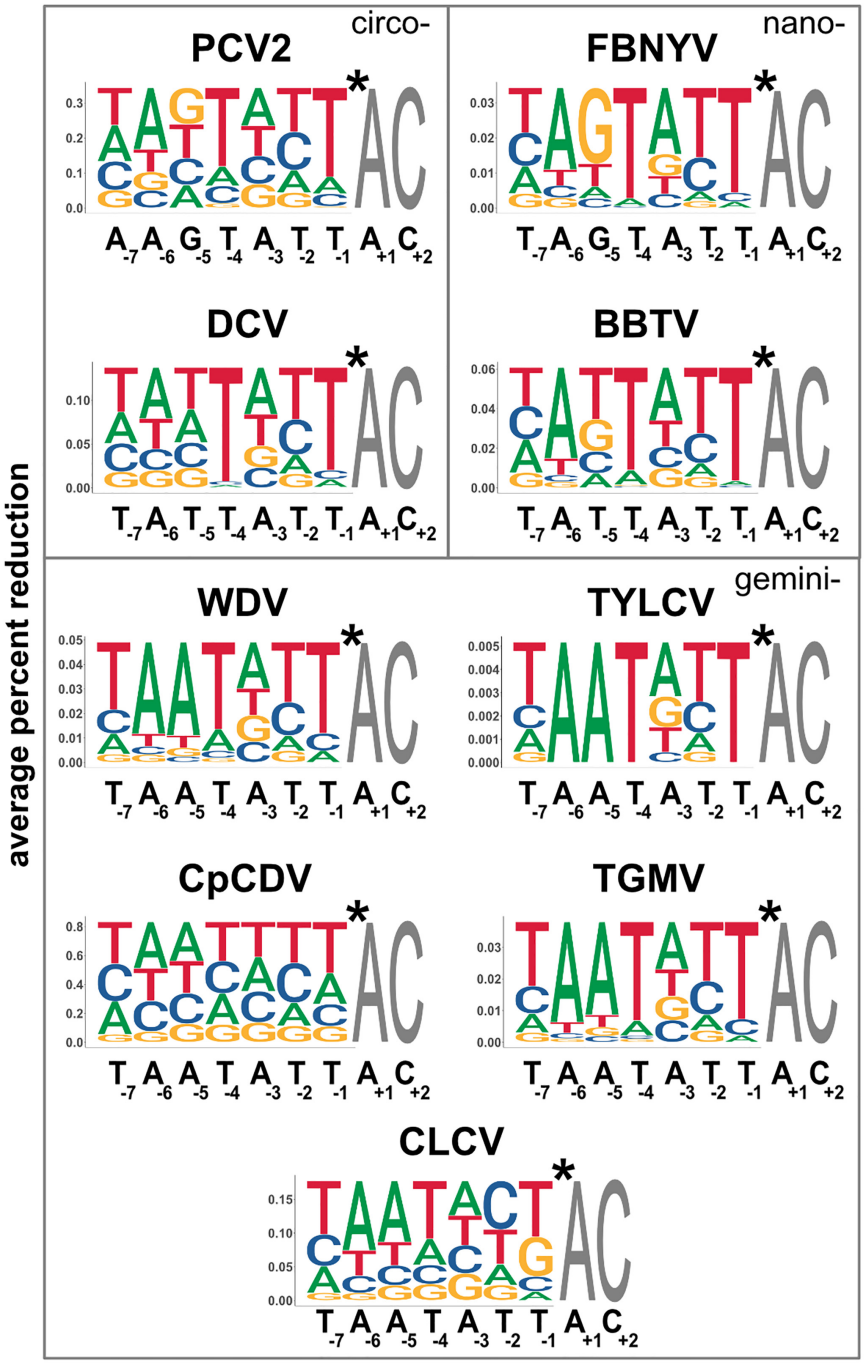
Weighted sequence logos generated from HUH-seq cleavage data. Weighted sequence logos for nine of the ten Reps based on percent reduction generated using ggseqlogo with values under 0.3 set to 0.0 in order to remove noise obtained from the HUH NGS cleavage assay. Heights are scaled to represent the average percent reduction of each base at each position when compared to the reference library. Sequences in black below each logo are the cognate nonanucleotide *ori* sequences from each respective virus. Asterisk denotes the cleavage site. Logos are organized by viral families as labeled inside the gray boxes.

We generated weighted sequence logos based on a *k*-mer reduction analysis with a threshold value of 0.3 or greater to reduce noise based on high confidence data guided by calculated adjusted *P*-values (FDR) (Figure 5, Supplementary Figure S5B). Percent reduction for each *k*-mer was calculated by comparing the normalized *k*-mer read counts for each Rep treatment in triplicate to *k*-mer read counts from the reference library. For each position in a Rep sequence logo, individual characters were scaled by the average percent reduction of all *k*-mers containing that character and position. Because every sequence permutation 5′ of the *nic* site is present in the 7N ssDNA library, sequence logos reveal Rep preferences for nucleotides relative to one another. The most obvious result is that the most preferred nucleotides in the first seven positions of sequence logos are nearly identical to the cognate nonanucleotide *ori* sequence found in each respective viral genome (Figure 5). While it is not surprising that the preferred target sequence is the same as the cognate nonanucleotide *ori* sequence cleaved *in vivo*, it also gives high confidence that HUH-seq can be used to quantitatively rank the *k*-mers cleaved by each Rep, analyze patterns that dictate these ssDNA recognition profiles, and further characterize differences between individual Reps.

Within each sequence logo, there are differentially preferred nucleotide positions. Positions T_−4_ and T_−1_ are almost unanimously the most preferred, while there is only slight preference for A and T at the −3 and −2 positions, respectively. There are also discernible trends between Reps from different families. For example, geminivirus Reps have a strong preference for adenine at the -5 position, whereas Reps from other families prefer thymine or guanine there (Figure 5). The y-axis scale of the weighted sequence logos also indicates the relative overall cleavage efficiency between Reps. For instance, PCV2 has a maximum average percent reduction of about 0.35 and cleaves roughly 10-fold more sequences than FBNYV, which has a maximum value of about 0.035. This indicates that PCV2 ssDNA recognition is more promiscuous than that of FBNYV. CpCDV has the highest maximum average percent reduction of 0.8 and has minimal nucleotide preference, indicating it has the most relaxed sequence specificity (Figure 5).

As controls, we included two WDV Rep treatments with lower protein concentrations and found that decreasing the amount of WDV Rep minimally affected specificity (Supplementary Figure S6A). To ensure cleavage was the only readout for this assay, we used an inactive WDV^Y106F^ Rep treatment, yet a small number of ‘cleaved’ *k*-mers were identified from the inactive treatment indicating that Rep binding may slightly contribute (Supplementary Figure S6C and S6D). Other considerations and caveats of HUH-seq analysis are discussed in supplemental information (Supplementary Note S4 and Supplementary Figure S6). Despite these caveats, HUH-seq is a robust method for profiling Rep specificity.

### Rep ssDNA recognition profiles corroborate structural observations

**Figure 6.**
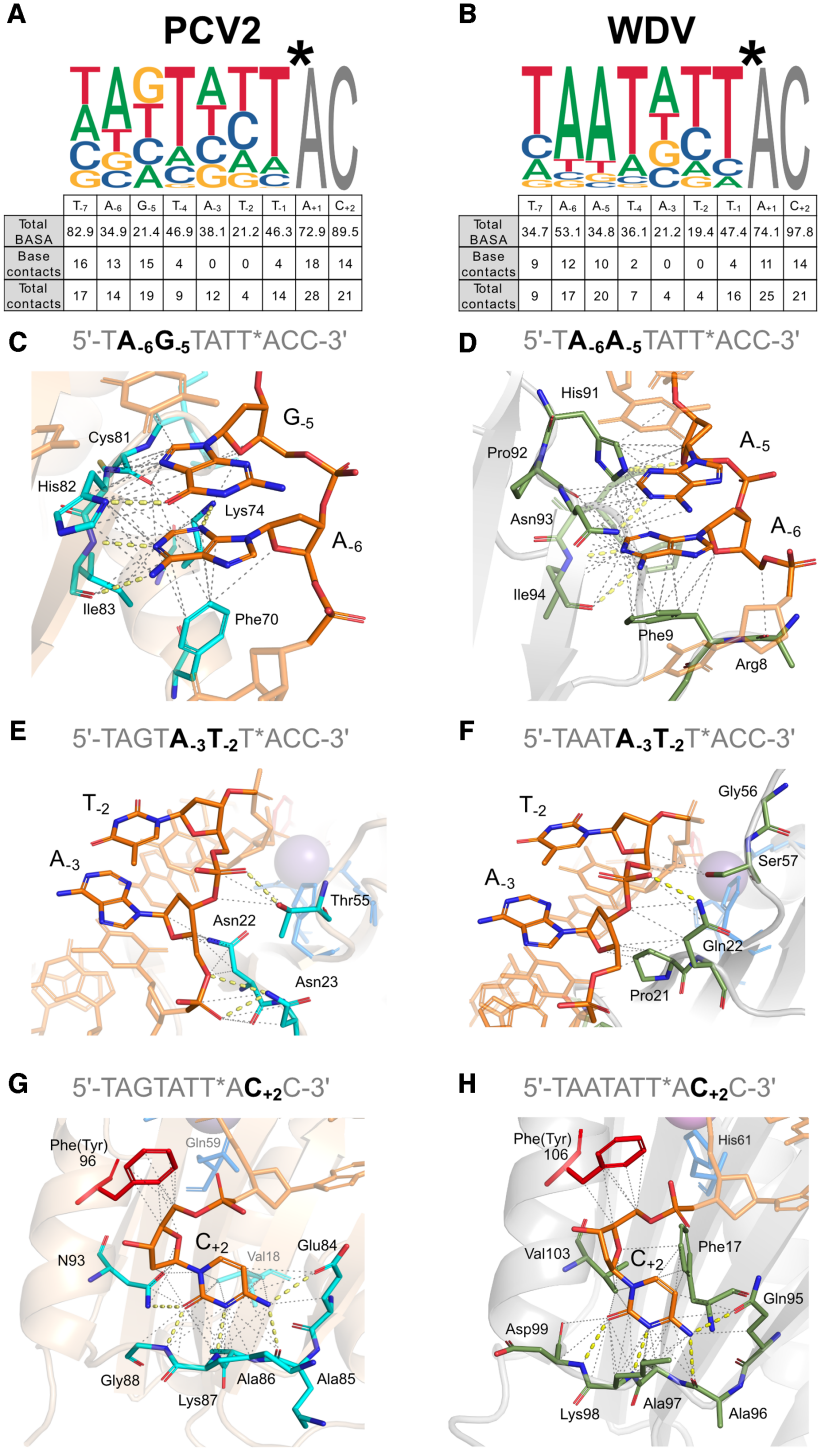
Comparison of Rep protein–DNA interactions and HUH-seq specificity profiles: (**A**), PCV2 + 10mer (6WDZ) and (**B**) WDV + 10mer (6WE0) BASA values and total number of protein-DNA contacts compared to weighted sequence logos from HUH-seq analysis. Both polar and van der Waals interactions are counted within 4 Å. (**C–H**) Atomic interactions between highlighted nucleotides within the bound 10-mers of PCV2 and WDV structures are shown with yellow dashes for polar contacts and gray dashes for van der Waals interactions within 4 Å. PCV2 cartoon is depicted in beige and residues interacting with DNA as sticks shown in cyan colored by atom. WDV cartoon is depicted in gray and residues interacting with DNA as sticks shown in green colored by atom. The PCV2 Phe96 and WDV Phe106 represent the catalytic tyrosine as sticks in red, divalent ion coordinating residues are in blue, and the manganese ion is a sphere in magenta. The 10-mer is shown as sticks in orange and colored by atom as highlighted in the panel.

Next, we quantified and assigned contributions of the ssDNA docking interface in the Rep structures to each nucleotide using DNAproDB by calculating the BASA as well as the total number of protein–DNA contacts (the sum of hydrogen bonds and Van der Waals interactions within 4 Å). Figure 6A and B summarizes the total BASA for and the total number of contacts with nucleotides corresponding to the cognate nonanucleotide *ori* sequence either with the entire nucleotide or the base only. These measurements in combination with the ssDNA recognition profiles of WDV and PCV2 were used to search for structural reasons why nucleotides in certain positions of the target sequence are conserved. A comprehensive table containing BASA and contact values of each of the three structures featured in this study is also provided (Supplementary Table S2). As expected, higher BASA values generally correlated to high numbers of contacts.

PCV2 bound 10-mer and WDV bound 10-mer structures have a similar total number of residues contacting DNA, 28 and 26 residues, respectively, and have a high concentration of base contacts and total contacts near the 5′ and 3′ termini of the 10-mers (Figure 6A and B). In Figure 6C–H, significant structural differences are highlighted between the contacts of nucleotides at different positions for both PCV2 (C, E and G) and WDV (D, F and H). A_−3_ and T_−2_ are the least conserved nucleotides at their indicated position. This is structurally consistent because there are zero contacts with the bases for both PCV2 and WDV indicating that specific nucleotides are not as preferred at these two positions because the interactions are exclusively with the ribose and phosphate of the nucleotides (Figure 6E and F). The 10-mer bound to PCV2 differs at position –5 between guanine and adenine with respect to the 10-mer bound to WDV. His91 and Asn93 of WDV facilitate polar contacts with A_−5_, which may give WDV more specificity at position –5, whereas there is only one polar contact with G_−5_ by His82 in the PCV2 structure, which results in less stringent specificity. (Figure 6C and D). Finally, in both structures C_+2_ dwells in a pocket of the protein surface with the highest BASA and total contact values (Figure 6G and H). Eight residues have contacts with C_+2_ in both structures, and five of these residues make up the last positions of the sDBM.

In contrast, T_−4_ is highly conserved as evident in all Rep ssDNA recognition profiles, but we observed only a marginal number of protein contacts with the base itself (Figure 6A and B). We hypothesize that the WC base pairing of T_−4_ with A_+1_ is a major contributor to the U-shaped conformation rather than contributing to sequence specificity via residue interactions with the base. Though Reps exhibit interactions with bases that contribute to specificity, it is clear from the ssDNA recognition profiles and minimal protein-DNA contacts at certain positions that Rep cleavage is also promiscuous, cleaving a wide range of target sequences. Taken together, there are two substantial contributors to Rep specificity: the first being the indirect readout of a given DNA sequence that adopts a conformation that fits into the groove of the Rep docking interface and the second being the direct readout of nucleotide bases through specific protein contacts.

### Discovering intrinsically orthogonal Rep target sequences using HUH-seq

During initial assessments of the HUH-seq analysis results, we noticed that there were individual target *k*-mers with drastically different log_2_FC values between different Rep protein treatments. This prompted us to ask whether we could identify pairs of *k*-mers that would allow us to selectively label two Reps in a single reaction mixture with unique oligos. For instance, *k*-mer, AGTCAAT (#2884) has a log_2_FC value of −3.44 for PCV2 and a near zero log_2_FC for every other Rep (Supplementary Figure S7). This result was validated using the *in vitro* HUH cleavage assay by reacting PCV2 Rep with a synthetic oligo containing this *k*-mer sequence. Indeed, only PCV2 formed a covalent adduct with the oligo harboring this target sequence (Supplementary Figure S7). Interestingly, this target sequence contains 4 substitutions with respect to the circovirus *ori* sequence at positions −6, −5, −4 and −2, again highlighting the promiscuous nature of Reps. This result revealed that searching for combinations of Reps and *k*-mers may result in the discovery of naturally occurring orthogonality despite apparent cross-reactivity.

**Figure 7.**
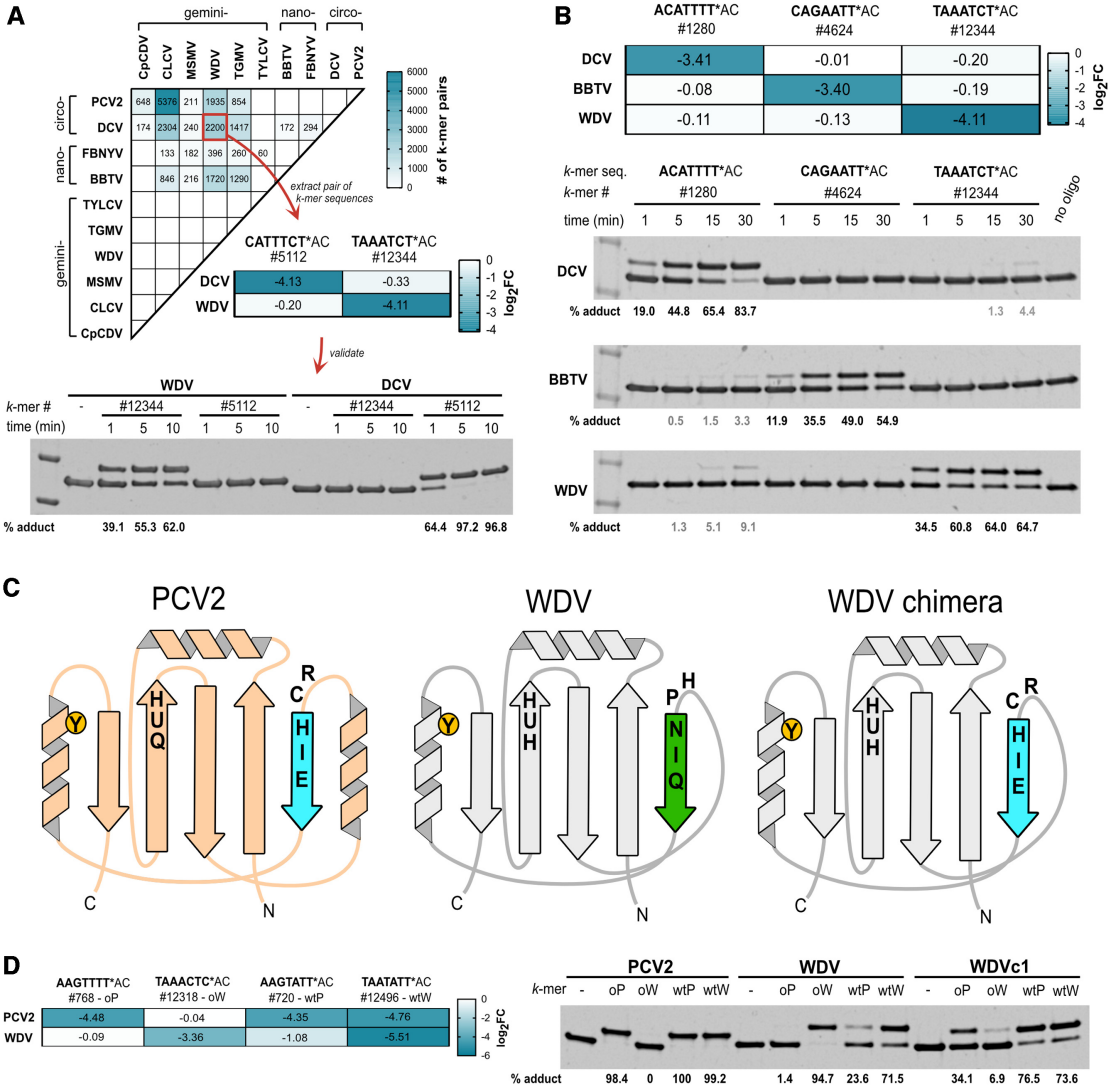
Discovery of orthogonal Rep target sequences and rational engineering of Rep specificity. (**A**) Heatmap displaying the number of *k*-mer pairs for a specific Reps set likely to be orthogonal using an asymmetric log_2_FC threshold based on values from HUH-seq analysis, blank cells indicate zero such *k*-mer pairs. The threshold values are set to log_2_FC values greater than -0.3 (indicating no *k*-mer cleavage) and log_2_FC values less than –3 (indicating high *k*-mer cleavage). Each cell of the heatmap represents the total number of possible *k*-mer pair combinations likely to be orthogonal for a particular set of two Reps. Sets are based on this asymmetric threshold in which the first Rep in the set has high cleavage of one *k*-mer in the pair and no cleavage of the other *k*-mer in the pair - vise versa for for the second Rep in the set, indicating orthogonality. As an example, one *k*-mer pair (#5112 and #12344) of the 2200 possible combinations indicated by the WDV vs. DCV cells were synthesized in the context of the flanking regions of the 7N ssDNA library, and cleavage orthogonality was validated using the *in vitro* HUH cleavage assay. Recombinant WDV and DCV were reacted under standard conditions over a short time course with synthetic oligos harboring *k*-mers sequences #5112 or #12344. Percent covalent adduct was calculated. (**B**) Set of three Reps and corresponding orthogonal set of three *k*-mer sequences as indicated by log_2_FC values from HUH-seq analysis. Oligos synthesized harboring *k*-mer sequences (#1280, #4624, #12344) were reacted with DCV, BBTV, and WDV recombinant Reps at room temperature with 1.5× molar excess oligo to Rep protein over a short time course. (**C**) A schematic illustrating the construction of the WDV chimera (WDVc1) containing the first five amino acids of the PCV2 sDBM. (**D**) The heatmap displays HUH-seq log_2_FC values for PCV2 and WDV reactivity with cognate nonanucleotide sequences (*k*-mers #720 and #12496) and a pair of *k*-mers (#768 and #12318) predicted to react orthogonally. The *k*-mers #720 (wtP), #12496 (wtW), (#768 (oP) and #12318 (oW) were synthesized in the context of the flanking sequences of the 7N ssDNA library and reacted in a 5x molar excess with PCV2, WDV, and WDV chimera (WDc1) recombinant protein for 30 min and 37°C.

To explore the possibility of naturally occurring orthogonality between two Reps, we wrote a script to extract pairs of *k*-mer sequences and Reps predicted to lack cross-reactivity based on log_2_FC values. Figure 7a displays a summary heatmap of the number of such *k*-mer pairs existing for every set of Rep pairs, based on threshold values of –0.3 log_2_FC and greater (likely forming no adduct) and –3.0 log_2_FC and lower (likely having high adduct formation). In one example, we identified the *k*-mer sequence, CATTTCT (#5112), in which DCV had a −4.13 log_2_FC and WDV had a −0.33 log_2_FC, and another *k*-mer sequence, TAAATCT (#12344), in which DCV had a –0.20 log_2_FC and WDV had a −4.11 log_2_FC, indicating orthogonality between DCV and WDV for these two *k*-mers. We validated this observation with an *in vitro* HUH cleavage assay including a short time course with 1, 5 and 10 min time points. DCV formed about 97% adduct with a synthetic oligo harboring *k*-mer #5112 over the course of 5 min, and WDV formed about 62% adduct with a synthetic oligo harboring *k*-mer #12344 over the course of 10 min. As expected, no cross-reactivity was observed between WDV with *k*-mer #5112 or DCV with *k*-mer #12344 (Figure 7A).

We next searched for triple orthogonal sets of Reps from our panel. As an example, the set containing *k*-mer sequences, #1280, #4624 and #12344, are predicted to react orthogonally with DCV, BBTV and WDV recombinant Reps, respectively, as indicated by log_2_FC values (Figure 7B). Similar to our method for validating double orthogonal sets, we tested the orthogonality of this set using the standard *in vitro* cleavage assay and calculated percent adduct formed with each combination of *k*-mer and Reps over a short time course. Expected orthogonality was achieved with over 50% covalent adduct formation after 30 min for each of the three Reps with 0–9% cross-reactivity identified (Figure 7B).

Notably, 23 of the 28 Rep sets from different viral families contained significant *k*-mer pairs likely to be orthogonal, yet there were no instances of orthogonal *k*-mer pairs for Reps derived from the same viral family (Figure 7A). Hence, the ssDNA binding moieties of Reps within the same family may be too similar to yield orthogonal adduct formation. This is a curious result in the case of DCV and BBTV where 294 potentially orthogonal *k*-mer pairs were identified, which are from different Rep families but recognize identical cognate nonanucleotide *ori* sequences (Figure 7A). This indicates that perhaps DCV and BBTV use different interactions to recognize the same cognate sequence allowing for divergent specificity at each nucleotide position. Indeed, six out of nine residues in the sDBM are different between DCV and BBTV Reps (Figure 2C). Using HUH-seq, we can pick out subtle differences in Rep specificity in order to extract double and triple orthogonal *k*-mers and Reps sets that can be used in multiplexed HUH-tag technologies. This could potentially negate the necessity for or create ways to use Reps in combination with the larger and slower relaxases or commercial fusion tags.

### Rational design of a WDV chimera confers PCV2-like sequence specificity

The identification of the sDBM, which we hypothesized was responsible for sequence specificity in Reps as well as the discovery of pairs of target sequences between two Reps that should not cross-react, inspired us to swap sDBM residues between two Reps and ask if we could alter sequence specificity. We swapped out the first five amino acids of the WDV sDBM for those of PCV2, creating a WDV chimera (WDVc1) as a proof-of-concept that Rep specificity could be altered by rational design in a predictable manner (Figure 7C). Because many of the amino acid side chains in both Rep structures have direct contacts with bases at the 5′ end of the ssDNA, we hypothesized WDVc1 would have sequence specificity more closely reflecting that of PCV2. First, we identified a pair of predicted target sequences for PCV2 and WDV using HUH-seq, where WDVc1 reacts with the *k*-mer #768 (oP), which was predicted to only react with PCV2, to a greater extent than *k*-mer #12318 (oW), which was predicted to only react with WDV (Figure 7D). Similar to PCV2, WDVc1 reacts robustly with the cognate nonameric sequence of PCV2, *k*-mer #720 (wtP), as well as the cognate nonameric sequence of WDV, *k*-mer #12496 (wtW), (Figure 7C). Thus, we show how the sDBM is a key feature of Reps that may be rationally engineered to predictably alter sequence specificity.

## DISCUSSION

We first determined the molecular basis of ssDNA recognition of viral Reps by solving crystal structures of viral Reps bound to ssDNA containing the cognate *ori* sequence trapped in the pre-cleavage conformation. Several apo structures of viral Reps in the absence of DNA have been reported (40–44) as well as parvovirus AAV5 Rep structures bound to distal auxiliary regions of dsDNA with the inverted terminal repeat (ITR) involved in rolling hairpin replication (45,46). However, the Rep structures presented here for the first time illuminate the interface responsible for specific ssDNA recognition necessary for ssDNA processing. The most striking feature of the ssDNA bound structures is the central role played by a motif we call the sDBM. This motif is highly conserved between members of the same viral Rep family but divergent between families, yet it maintains its key function of binding ssDNA for cleavage across the HUH endonuclease superfamily (Figure 3, Supplementary Figure S3). The sDBM motif partially overlaps with a previously identified ∼20 amino acid long motif specific to the geminivirus family called the GRS, which was suggested to interact with DNA via mutagenesis studies (37). The sDBM facilitates two recognition modes: the first mode is indirect, whereby target sequences that have the propensity to adopt a U-shaped conformation (e.g. via base-pairing and stacking) fit into the groove of the Rep docking interface, and the second mode is direct, whereby the Rep provides specific protein-ssDNA contacts conferring specificity for ssDNA based on amino acid sequence. The combination of these modes accounts for the semi-promiscuous specificity of viral Reps.

Looking more broadly at the Rep-DNA interactions in the context of the HUH-endonuclease superfamily, the sDBM is apparently a ubiquitous motif contributing to DNA binding and recognition. This is illustrated by available relaxase and transposase structures captured in the pre-cleavage state (17,28–30,47). In Reps, the sDBM is located in the middle of the structure and consists of the fourth beta strand and a portion of the preceding loop. In relaxases, however, the sDBM is located at the extreme N-terminus due to the circular permutation of relaxases with respect to viral Reps and plays a major role in forming specific contacts especially with nucleotides bound in deep pockets of the protein surface. Transposases, like relaxases, bind a hairpin sequence distal from the cleavage site. However, recognition of the cleavage site occurs by both the protein and a short guide sequence near the stem of the hairpin. In the ternary structure of the IS*608* TnpA transposase (PDB ID: 6FI8) in complex with its hairpin ‘imperfect palindrome’ sequence and a short oligonucleotide spanning the cleavage site, the –1 and +1 bases form base pairing interactions with the guide sequence of the hairpin DNA bridging the two distant sections of DNA to form a U-shape (47,48). This ‘*trans*’ U-shape conformation is primed much in the same way viral Reps bend the ssDNA using the sDBM, most notably by the bridging Phe112 that stacks with C_+1_ (Supplementary Figure S3C). Other amino acids of the sDBM also bind to the guide DNA sequence and seem to play a greater role in conformation priming than direct recognition; though DNA bases extending downstream the +1 position are missing in the structure, preventing further discussion of the role of the transposase sDBM in specificity.

In nature, there are several reasons why Rep specificity could be more promiscuous than that of relaxases. If conjugative plasmid transfer occurs at an erroneous origin, it could result in catastrophic consequence for the host's fitness; whereas, there is little selective pressure for a virus to initiate replication at a very specific sequence due to the small number of sequences within a sub-5 kb genome (32). Relaxases may also have higher specificity for the DNA sequence 5′ of the *nic* site for more efficient catalysis of rejoining of the free 3′OH of the DNA post-transfer, whereas RCR resolution would likely require a second dimerizing Rep for termination (49). It should also be noted that Rep specificity could also simply be constrained by a smaller interface surface area due to limited gene size (50).

Characterization of the Rep/ssDNA interface provides a platform to model other Rep ssDNA interfaces as well as an avenue to explore disruption of the interface for antiviral treatments of plant and human Rep-mediated viral infections (51). Billions of dollars worldwide are lost in agriculture every year from the decimation of crops such as tomatoes, cassava, cotton, and beans by geminivirus infection (52). In a human disease context, parvovirus B19 human infections can lead to serious or fatal outcomes for a fetus (53,54) and are associated with autoimmune diseases in adults (21,22). This has sparked treatment and vaccine development (55), however present antiviral strategies, both viral protein interfering and gene silencing approaches, are either minimally effective or are eventually subverted by conferred resistance from a rapidly evolving viral genome (51,56–60). Development of antivirals specifically targeting the ssDNA binding of Reps could more effectively retain long-term resistance.

The Rep structures revealed highly conserved protein-DNA interfaces with subtle differences that prompted us to ask whether Reps within families and from different families differentially tolerate mutations of the cognate *ori* sequence. One reported relatively high throughput strategy for querying key nucleotides in bacterial conjugation mediated by the relaxase TrwC used saturation mutagenesis in concert with a functional DNA-transfer readout (32), which is a readout incompatible with Reps. Other NGS-based ssDNA recognition approaches, for example of cytosine deaminases (61) or DNA aptamer-binding protein targets (62), also use direct sequencing readout methodology. However, a direct readout of Rep cleavage is technically challenging due to the need to amplify physically separated cleaved DNA molecules and covalent attachment of the Rep to the new 5′ end of the cleaved molecules. Instead, HUH-seq, allows for the quantitative readout of Rep cleavage specificity using a ssDNA library with a subtractive, or reduction, readout.

Excitingly, we found HUH-seq can be used to distinguish subtle differences between Rep nucleotide preferences despite overall lack of specificity, so much so that intrinsic orthogonality between non-cognate target sequences can be extrapolated between Reps from different, yet closely related, viral families with highly similar or even the same cognate nonanucleotide *ori* sequences. Intrinsic orthogonality between Rep families demonstrates the feasibility of using Reps in multiplexing applications without the need for protein engineering, despite their apparent promiscuity. Moreover, there are currently 10 additional viral Rep families yet to be explored with HUH-seq (50), meaning that multiplexing could be expanded to up to 13 Reps in a given system (i.e. have 13 HUH-fusions in an application such as DNA barcoding of proteins of interest and add 13 DNA barcodes that should specifically react with only one given Rep HUH-tag). DNA-tagging is the basis of established technologies such as proximity ligation (63) and DNA-PAINT super-resolution imaging (64) as well as emerging applications such as multiplexed single-cell proteomics (65), and optics free DNA microscopy (66), where parallel tracking of proteins occurs using NGS. It is of note that HUH-fusions would allow conjugation of oligos to ScFv's and nanobodies, which could expand the utility of many of these applications which utilize oligo-conjugated antibodies. Because HUH-tag linkages are specific and covalent, can occur intra- or extracellularly without additional reagents, and are now multiplexable, they are ideal fusion tags for these applications.

We foresee the utility of the simple HUH-seq approach, with minor alterations, for sensitive detection of sequence specificity profiles for enzymes such as dsDNA nucleases by simply using a dsDNA library, RNA-cleaving enzymes by adding a single reverse transcriptase step, or site-specific nucleotide modifying enzymes by relying on a covalent modification that blocks PCR amplification. The existing high-diversity library methods used to determine the dsDNA specificity of zinc finger nucleases (67), Cas9 (68), transcription activator-like effector nucleases (TALENs) (69), and other restriction enzymes (70) are powerful and direct cleavage readout approaches, however they require a number of extra library preparation steps and may be limited to only dsDNA libraries. Additionally, if sequence binding, rather than cleavage, could be optimized as a readout, HUH-seq could be developed as a facile alternative method to approaches such as SELEX-seq (71) and could determine binding sequence preference of shorter DNA binding motifs. Lastly, an HUH-seq screen including a broad range of Rep-encoding organisms may yield crucial evidence for more accurate lineage classification, which is continually being restructured most notably because of the exponential discovery rate of unclassified circular ssDNA viruses (72). The *rep* gene is an indispensable component of lineage analysis (73,74) and a combination of *rep* gene structure and sequence identity along with Rep cleavage specificity may lead to rapid and more accurate classification.

While subtle but specific family differences in DNA recognition coupled with HUH-seq permits modest viral Rep multiplexing, expanded multiplexing capability could be achieved by engineering the protein to recognize designer DNA sequences. Engineering one HUH-endonuclease to react with another HUH-endonuclease target sequence has been demonstrated for AAV but involved swapping large protein domains (75). Similarly, a double mutant of the TraI relaxase that conjugates the F plasmid was able to switch specificity to the related R100 plasmid target sequence, though the engineering was performed by testing and mutating all distinct amino acids residues between the two relaxases (76). We have shown in an elegant example of rational engineering that by simply mutating four amino acids within the sDBM of WDV and PCV2, specificity can be predictably altered. This approach was made possible not only by structural insights, but also because we can identify intrinsically orthogonal target sequences that would react specifically with each Rep using HUH-seq. Predictable altering of ssDNA specificity by targeting the residue composition of the sDBM either by rational design or directed evolution could motivate development of engineered HUH-tags with defined sequence specificity to facilitate massively parallel Rep-based applications or to facilitate integration of AAV Reps into desirable sites in the genome.

There are several potential improvements on our studies. While these Rep-ssDNA structures are likely highly representative of RCR mediating ssDNA viruses, parvovirus Reps exhibit some sequence and structural differences. For example, the sDBM of AAV Reps include an additional charged loop that may provide an added level of specificity or contribute to binding of ITR hairpins (46). Elucidating the exact ssDNA binding mode of action of parvovirus Reps would provide more specific information for engineering specificity for gene integration applications or designing Rep-targeting antivirals for human disease causing viruses. HUH-seq is limited by the diversity size of the library; however, as NGS read capacity, speed, and cost-effectiveness increase, along with computational processing and data storage, library size may become a negligible shortcoming of HUH-seq. While we use a limited diversity library of 7 randomized nucleotides in this study, it still allowed us to interrogate specificity of many Reps at once in addition to allowing a number of controls in a single HiSeq sequencing lane. A more complete Rep specificity profile using an expanded sequence library is possible using HUH-seq, however this greatly limits the number of Reps and controls that can be used in a single NGS sequencing lane. Using multiple sequencing runs and lanes would be a simple solution to this issue though would dramatically increase cost. Additionally, given that the HUH-seq inactive WDV^Y106F^ mutant control revealed *k*-mers with significant percent reduction over the reference library, a more stringent denaturation step may ensure cleavage is the only readout rather than both binding and cleavage (Supplementary Figure 6C and D). Finally, unknown binding and cleavage kinetic factors may differ for each Rep and could be convoluting our ability to compare specificity. Ascertaining a full kinetic profile of each of these steps may give a better comparative picture of sequence specificity.

Together the combination of structural and NGS approaches demonstrate that viral Reps, with desirable size and reaction efficiency but low apparent sequence specificity, can be exploited in multiplexing applications by engineering DNA target sequences and protein sequences. These findings will drive further studies into engineering HUH-endonuclease recognition of ssDNA and expanded application of HUH-endonucleases as HUH-tags.

## DATA AVAILABILITY

Co-crystal structure coordinates and structure factors of PCV2^Y96F^ + 10-mer, WDV^Y106F^ + 10-mer, and WDV^Y106F^ + 8-mer complexes were deposited with accession codes 6WDZ, 6WE0, and 6WE1, respectively, in the Protein Data Base (PDB).

## Supplementary Material

gkaa1248_Supplemental_Files
