# Supplementary material for: Molecular underpinnings of ssDNA specificity by Rep HUH-endonucleases and implications for HUH-tag multiplexing and engineering
Source: Nucleic Acids Res. 2021 Jan 7;49(2):1046–64. doi: 10.1093/nar/gkaa1248 (PMC7826260; doi:10.1093/nar/gkaa1248)
Supplement: gkaa1248_Supplemental_Files [file gkaa1248_supplemental_files.zip › 20201002_NAR_SI_submission_revision_v2.pdf]

Molecular underpinnings of ssDNA specificity by Rep HUH-endonucleases and implications for HUH-tag multiplexing and engineering

Supplementary Information

Supplementary Table S1: Panel of Reps

**Rep** (aa range)	**Viral Species**	**Viral Family**	**Accession**	**5' Stem sequence**	**Loop sequence**	**3' Stem sequence**	**Loop length (bp)**	**Stem length (bp)**
PCV2 (2-116)	*Porcine circovirus 2*	*Circoviridae*	NC_005148	GAAGTGCGCTG	T**AAGTATT*AC**	CAGCGCACTTC	10	11
DCV (1-111)	*Muscovy duck circovirus*	*Circoviridae*	KR491947	ACAAGCGCCG	TTA**TATTATT*AC**	CGGCGCTTGT	12	10
FBNYV (1-98)	*Faba bean necrotic yellows virus*	*Nanoviridae*	NC_024457	CTGGGGCGGGG	CT**TAGTATT*AC**	CCCCGCCCCAG	11	11
BBTV (1-94)	*Banana bunch top virus*	*Nanoviridae*	KM607712	AGCGCTGGGG	CT**TATTATT*AC**	CCCCAGCGCT	11	10
WDV (1-137)	*Wheat dwarf virus*	*Geminiviridae*	AJ311031	CCACGCGGG	TTA**TAATATT*AC**	CCCGCGTGG	12	9
TYLCV (1-136)	*Tomato yellow leaf curl virus*	*Geminiviridae*	AJ489258	GCGGCCATCCG	TA**TAATATT*AC**	CGGATGGCCGC	11	11
CpCDV (1-124)	*Chickpea chlorotic dwarf virus*	*Geminiviridae*	LN865163	GCCACGCCG	AAAT**TAATATT*AC**	CGGCGTGGC	13	9
MSMV (1-117)	*Maize striate mosaic virus*	*Geminiviridae*	MF167301	AGATGGTGCGC	ATAA**TAATATT*AC**	GCGCACCATCT	13	11
CLCV (1-116)	*Cabbage leaf curl virus*	*Geminiviridae*	DQ178612	GCGGCCATCCG	CAA**TAATATT*AC**	CGGATGGCCGC	12	11
TGMV (1-124)	*Tomato golden mosaic virus*	*Geminiviridae*	JF694490	GCGGCCATCCG	TT**TAATATT*AC**	CGGATGGCCGC	11	11

Supplementary Figure S1: Simulated annealed omit map for Rep crystal structures

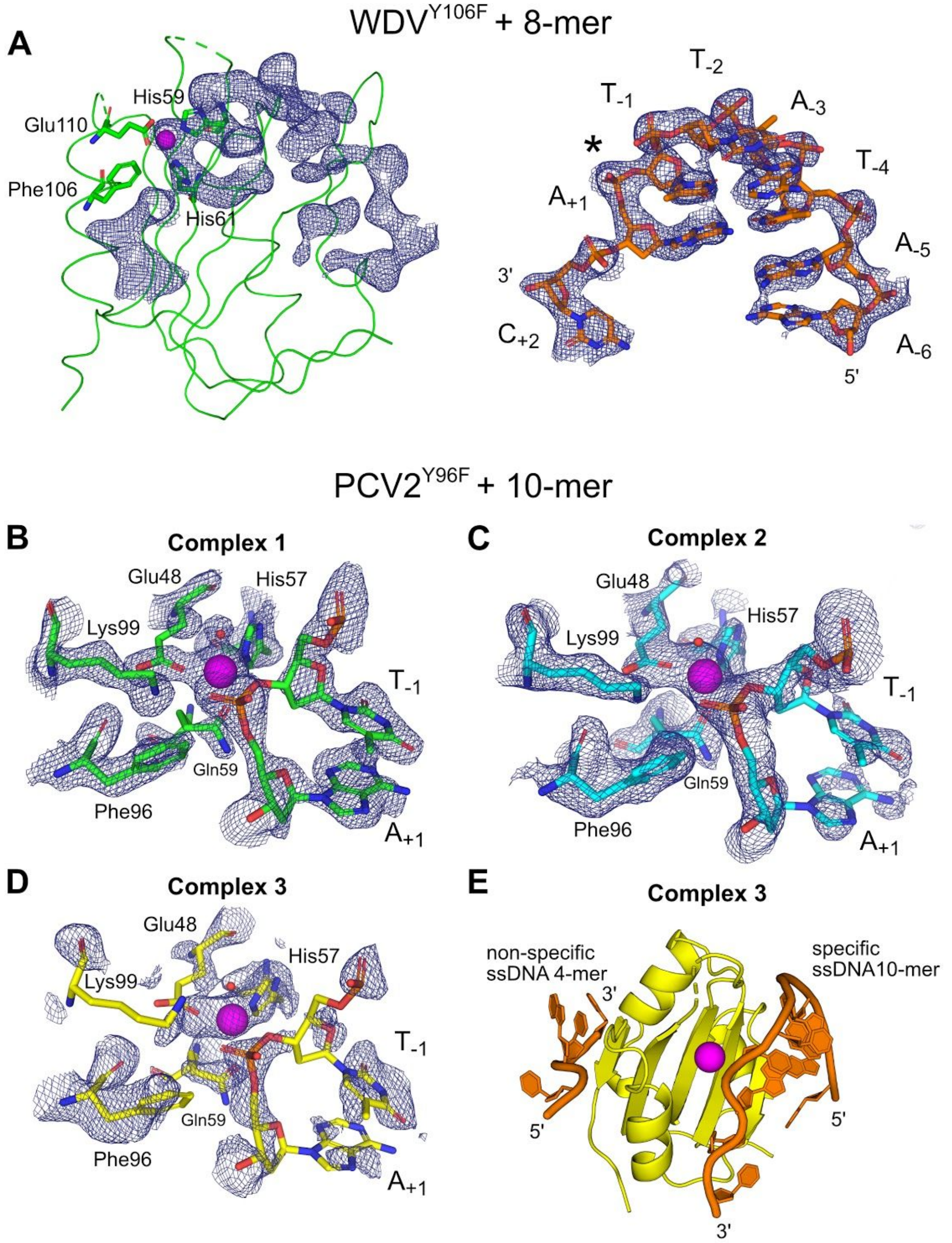

Figure S1: A, A 2mFo-DFc simulated annealed omit map represented as a dark blue mesh, σ = 1.5, was generated using coordinates for apo WDV^{Y106F} Rep as a model. A continuous electron density tunnel near the active site (side chains are green sticks and manganese as a magenta sphere) and WDV protein (green cartoon loops) surface was used to model in a single stranded DNA shown as orange sticks colored by element. The asterisk denotes the position of the scissile phosphate. **B-D,** A 2mFo-DFc simulated annealed omit map represented as a dark blue mesh, σ = 1.5, was generated using PCV2+10-mer (6WDZ) coordinates with removed active site residues (Glu48, His57, Gln59, Phe96, and Lys99), magnesium, and T_{-1} and A_{+1} nucleotides from each of the three complexes in the asymmetric unit. Complex 1 (green) and Complex 2 (cyan) have well-defined density, while Complex 3 (yellow) has poorly defined electron density. **E,** Cartoon of PCV2 Complex 3 bound to both the 10-mer and a modeled tetranucleotide portion of the 10-mer (orange), which is non-specifically bound.

Supplementary Note S1

The PCV2^{Y96F} + 10-mer active site contains an HUQ motif rather than an HUH motif along with a structurally conserved Glu48 that coordinates a manganese ion. Octahedral coordination of the ion is completed by an adjacent water molecule, which may be positioned by Arg54 or Glu100, and the O1 and O3’ of the scissile phosphate. A sequence conserved lysine, Lys99, may act as a general base and deprotonate the catalytic tyrosine poised near Phe96. The WDV^{Y106F} + 10-mer active site reveals identical coordination of manganese by the HUH motif and scissile phosphate. In contrast, Glu110 is used to coordinate the metal ion instead of the predicted Glu49 residue. This seems to result in shifting Lys109 out of position to act as a general base. It is unknown whether this coordination is a crystallographic artifact or if geminivirus Reps use a different cleavage mechanism that does not rely on a general base to activate the tyrosine. Rep and relaxase active sites are highly similar and perform the same general DNA cleavage mechanisms, using divalent metal ion coordination of O1 and O3’ of the scissile phosphate. This polarizes the partial positive charge of the phosphate catalyzing a nucleophilic attack by an adjacent active residue, though relaxases use a second conserved tyrosine for the DNA re-joining reaction (1).

Supplementary Figure S2: DNAproDB analysis of co-crystal Rep structures

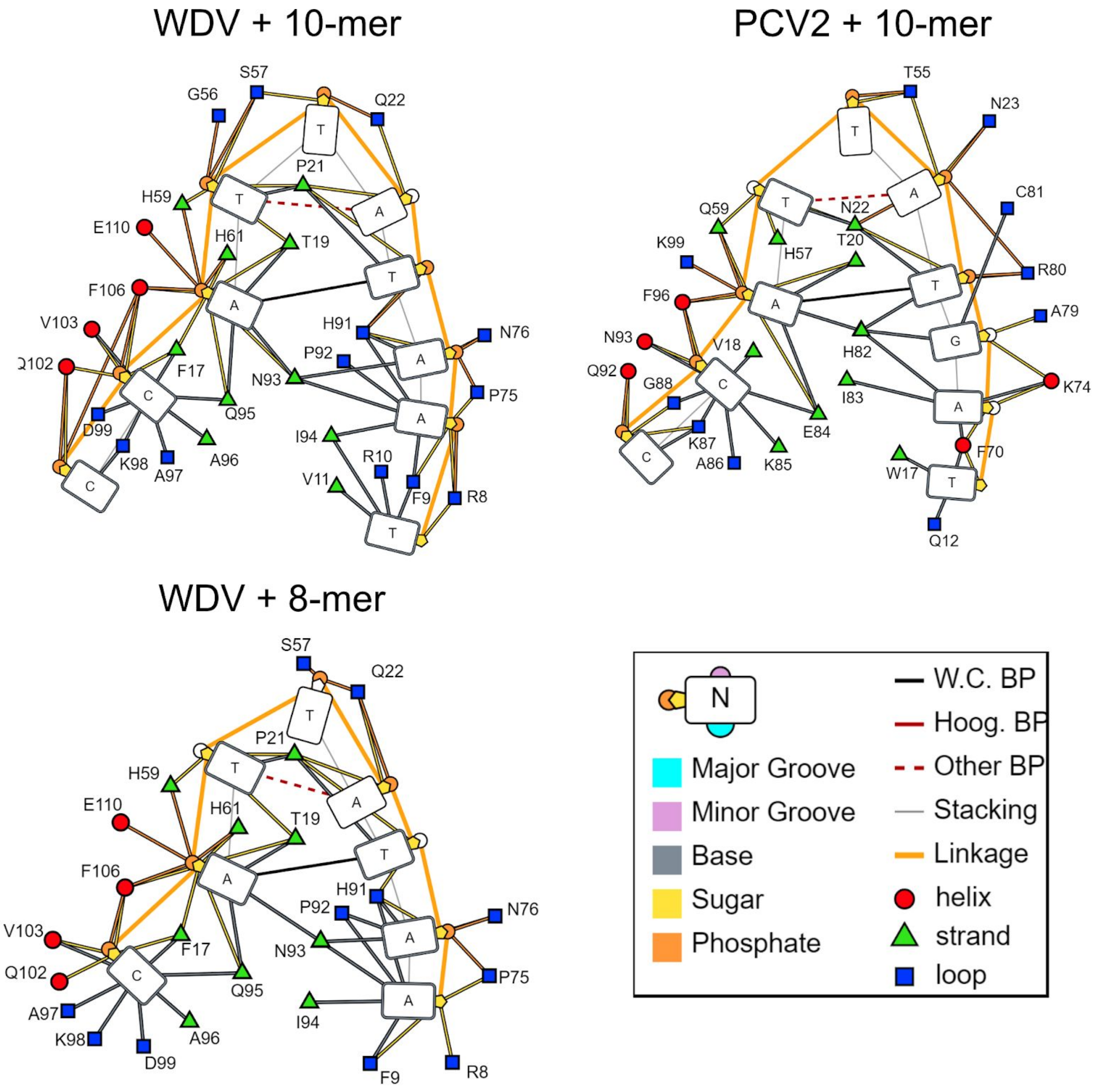

Figure S2: DNAproDB analysis of PCV2^{Y96F} and WDV^{Y106F} bound to a 10-mer and WDV^{Y106F} bound to 8-mer structures using default settings displaying contacts within 4 Å of the nucleotide backbone, sugar, and phosphate. Figure annotation and visualizations are taken directly from analysis software.

Supplementary Figure S3: The sDBM is a conserved ssDNA binding motif spanning HUH endonuclease classes

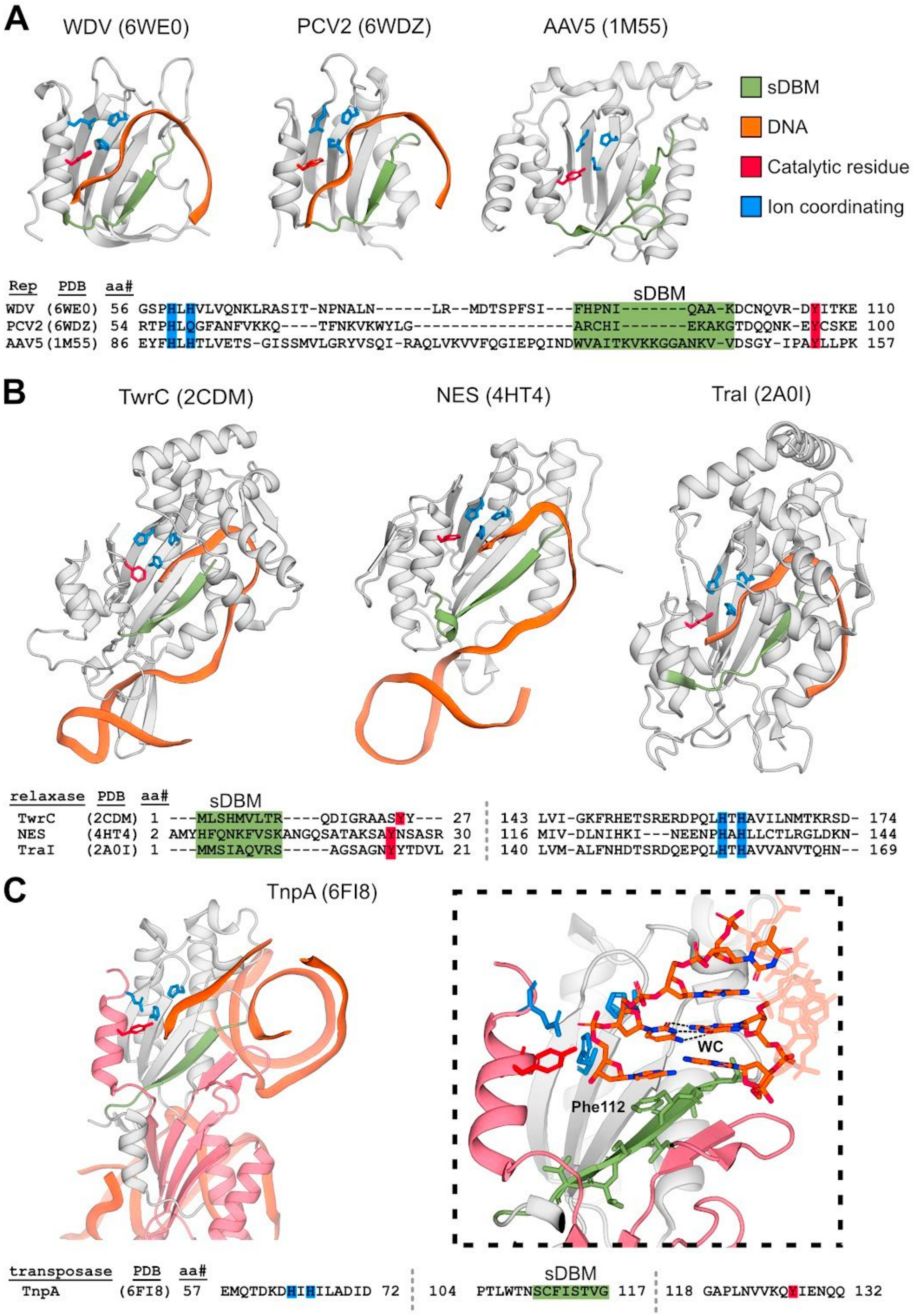

Figure S3: Structural alignment using PROMALS3D, using indicated PDB ID structures, and structural cartoon renderings highlight the relative positions of the sDBM (green) in **A,** β4 for Reps and **B,** β1 for relaxases. **C**, The sDBM in β4 for transposase TnpA aids stabilization of a "trans" U-shape and alpha helix containing the catalytic tyrosine is domain swapped from a homodimer (pink) Sections of the structural alignment consisting of the sDBM, the catalytic residue highlighted red, and the HUH-motif highlighted in blue are shown. Structures of protein-DNA complexes were used where available. DNA is shown as orange cartoon ribbon following C3 in the ribose. Any bound metal ions are hidden.

Supplementary Note S2

Sequential truncations of this geminivirus *ori* sequence of either the 5' end or 3' end indicated that at most, the nonameric sequence is necessary for sufficient cleavage activity because all ten Reps produced high adduct formation when the full nonameric sequence was retained in the target oligo. Perhaps the position -7 nucleotide is not necessary, because adduct formation was nearly identical in the absence of this nucleotide. Strikingly, CpCDV, and to a smaller extent PCV2 and CLCV, retain cleavage activity when only nucleotides in positions -2 through +2 are present ($T_{-2}T_{-1}$*$A_{+1}C_{+2}$). In contrast, TYLCV retained cleavage activity only when 8 of the 9 positions are retained in the target sequence, negating position -7 ($A_{-6}A_{-5}T_{-4}A_{-3}T_{-2}T_{-1}$*$A_{+1}C_{+2}$).

Single substitutions along the target sequence had no broad effect on the geminivirus Reps, except TYLCV activity was hampered by substitutions at positions -6 through +2. The nanovirus Reps, FBNYV and BBTV, have nearly identical target sequence profiles to that of TYLCV but tolerate both a transition and transversion at position -5. Interestingly, several double substitutions flanking the cleavage site of the target sequence were largely tolerated by geminivirus Reps, again with the exception of TYLCV, but cleavage of these sequences by circovirus and nanovirus Reps were abrogated.

Supplementary Figure S4: Standard *in vitro* cleavage assay with Rep panel and oligo library

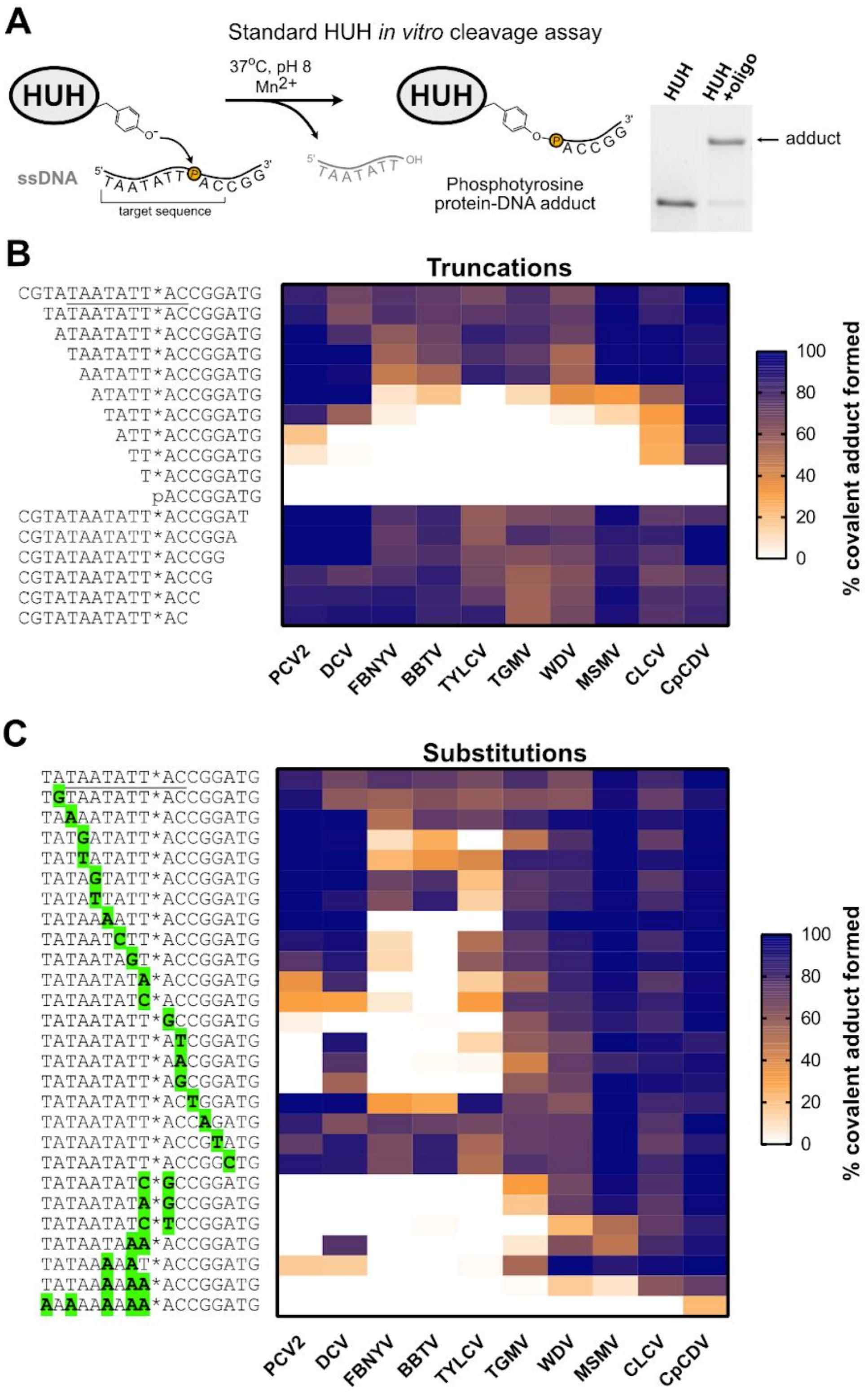

Figure S4: A, The in vitro HUH cleavage assay schematic where the catalytic tyrosine forms a covalent adduct with the phosphate at the +1 position scissile phosphate in the target sequence of a synthetic oligo. The phosphotyrosine adduct is stable under denaturing conditions evident by an upward shift from SDS-PAGE analysis. **B,** The truncations heatmap displays Rep covalent adduct formation with sequential truncations of synthetic DNA oligos harboring partial geminivirus *ori* target sequence with the cognate nonanucleotide *ori* sequence underlined. **C**, The substitutions heatmap displays HUH-endonuclease covalent adduct formation with single or multiple substitutions within synthetic DNA oligos harboring the geminivirus *ori* target sequence, either 26-nt or 17-nt in length. The conjugation reaction is carried out under the in vitro HUH reaction conditions with a 1:10 Rep:oligo ratio for 30 minutes. Each cell reflects a percent covalent adduct formation for a single reaction.

Supplementary Figure S5: Distribution of *k*-mer representation and HUH-seq data confidence

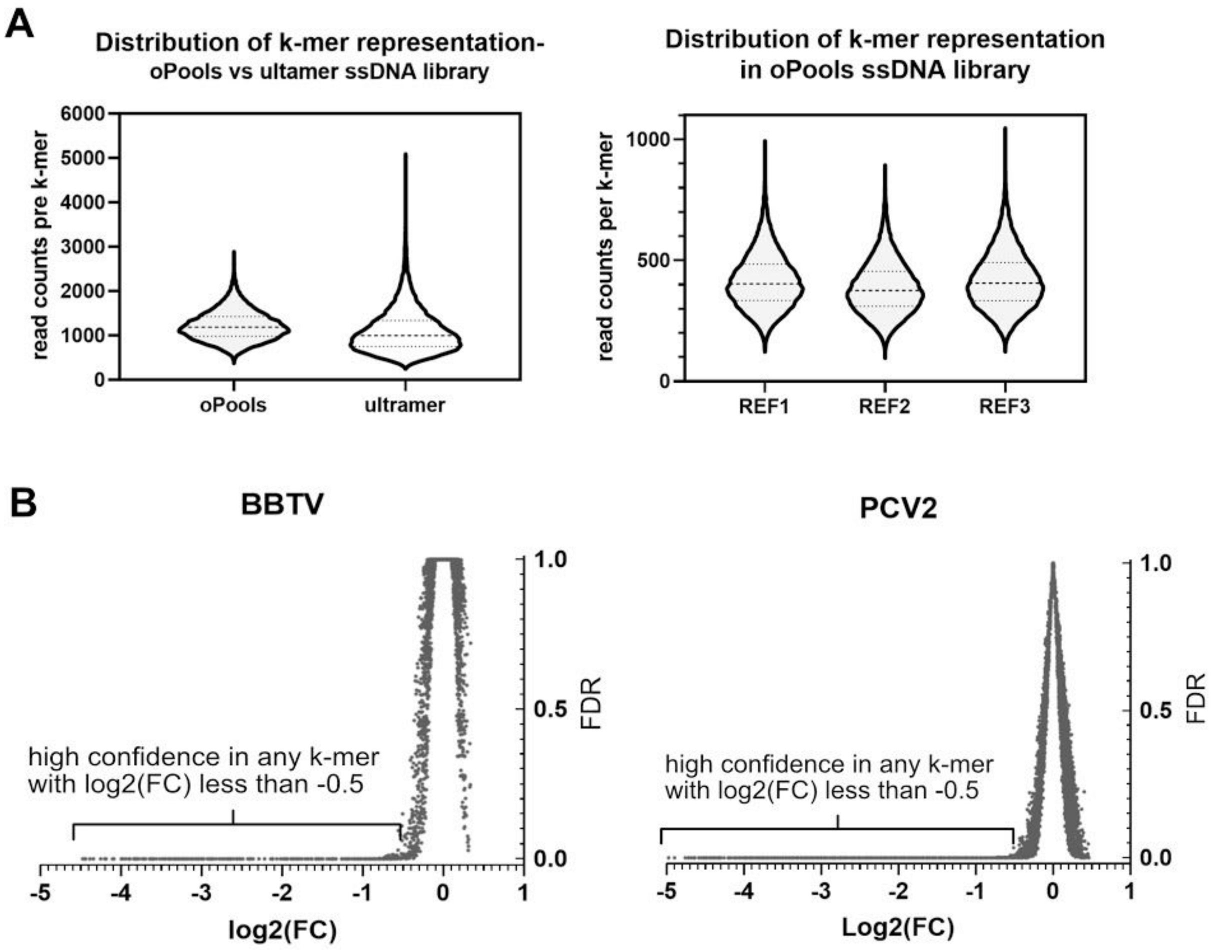

Figure S5: **A**, Violin plot depicting the distribution of all 16,384 *k*-mers in the oPools generated 7N ssDNA library versus the ultramer generated 7N ssDNA library based on a sum of all read counts at each *k*-mer position from three untreated replicates and on the left depicting distribution of all 16,384 *k*-mers present in each of the untreated reference (REF) replicates. **B**, Two representative scatter plots (BBTV and PCV2 Reps) of Log_2FC values versus FDR (adjusted p-value) highlighting the high confidence in any k-mer with a Log_2FC value less than -0.5 was cleaved by the Reps.

Supplementary Note S3:

Ideally, every sequence in the library must be at a high read count (>100 reads) since HUH-seq is a read count reduction based assay, so we limited the library size (SI Eq. 1) based on the total read counts provided by the HiSeq platform. We reasoned the size of the library, number of treatments, number of replicates, total read counts desired per *k*-mer, and percent of PhiX spike-in would all contribute to how large a library could be used (SI Eq. 2). We settled on creating a ssDNA library with 7 randomized nucleotides, which would produce on average 415 read counts per *k*-mer for 24 samples in triplicate (Eq. 1 and 2.).

Supplementary Equations 1 and 2:

Eq. 1: Determining total kmers in ssDNA library

$$(\text{\# of nucleotide options})^{(\text{\# of positions in seq.})} = \text{total kmers in ssDNA library}$$

Ex. 1:

$$(\text{4 nucleotides})^{(\text{7 positions})} = \text{16,384 kmers in ssDNA library}$$

Eq. 2: Estimating average read count per kmer for specified sequencing platform

$$\frac{(\text{total read count output}) - 30\%\ \text{PhiX}}{(\text{\# kmers in lib.})(\text{\# of smpls.} + 1\ \text{ref.})(\text{\# of replicates})} = \text{average read count per kmer}$$

Ex. 2:

$$\frac{(\text{700,000,000 reads, paired-end HiSeq}) - 30\%\ \text{PhiX}}{(\text{16,384 kmers})(\text{23 smpls.} + 1\ \text{ref.})(\text{3 replicates})} = \text{\textasciitilde 415 read counts per kmer}$$

Equation 1: Determines the number of total *k*-mers of user specified length in the synthetic ssDNA library dictated by the number of nucleotide options and number of randomized positions. Equation 2: is used to estimate the average read count per *k*-mer dictated by estimated total read count given by a NGS platform, minus the recommended 30% PhiX spike in, all divided by the number of kmers in the ssDNA

library, number of HUH-endonuclease samples and reference sample, and the number of replicates. This can be used to help determine which NGS platform will give sufficient read counts per *k*-mer.

Supplementary Note S4: HUH-seq cleavage assay caveats and controls

Lower concentrations of WDV had minimal impact on the ssDNA recognition profile of WDV, yet the lower maximum average percent reduction values correspond to fewer *k*-mer sequences being cleaved (Supplementary Figure S6A). Removing SUMO from WDV minimally affected the ssDNA recognition profiles as well (Supplemental Figure S6B). Interestingly, the inactive WDV^{Y106F} mutant control revealed *k*-mers with significant percent reduction over the reference library but to a much lesser extent than wild-type WDV. We have verified that this inactive WDV mutant does not cleave the 26-nt geminivirus nonanucleotide *ori* sequence oligo using the standard in vitro cleavage assay (Supplementary Figure S6C and S6D). We reasoned that the WDV^{Y106F} is still able to tightly bind the preferred target sequence and decrease the read count perhaps by partially blocking amplification of sequences bound to Rep rather than physical separation of the PBS sites due to cleavage. This result indicates that decreased read counts may be a consequence of both Rep binding and cleavage at a detectable yet much lower rate. Further, the ssDNA recognition profile generated for WDV^{y106F} is almost identical to that of WT WDV. We could refine this assay in the future with a more effective Rep inactivation step ensuring that cleavage is the only readout for the assay.

Another caveat we noticed is that the MSMV sequence logo did not show a preferred target sequence profile similar to that of the cognate nonanucleotide *ori* sequence. We presume that MSMV had a high cleavage rate in the constant region of the 7N ssDNA library somewhere near the 5' end of the randomized region resulting in a largely random sequence logo (Supplementary Figure S6E). Optimizing the sequence of flanking constant regions of the 7N ssDNA library to limit cleavage in this region may allow us to generate an accurate target sequence cleavage profile of MSMV in the future. Simple optimization could eliminate these caveats, though it would give uncritical advantages.

Supplementary Figure S6: Effects of enzyme concentration dependence, tag-less enzyme, and inactive enzyme on HUH-seq readout.

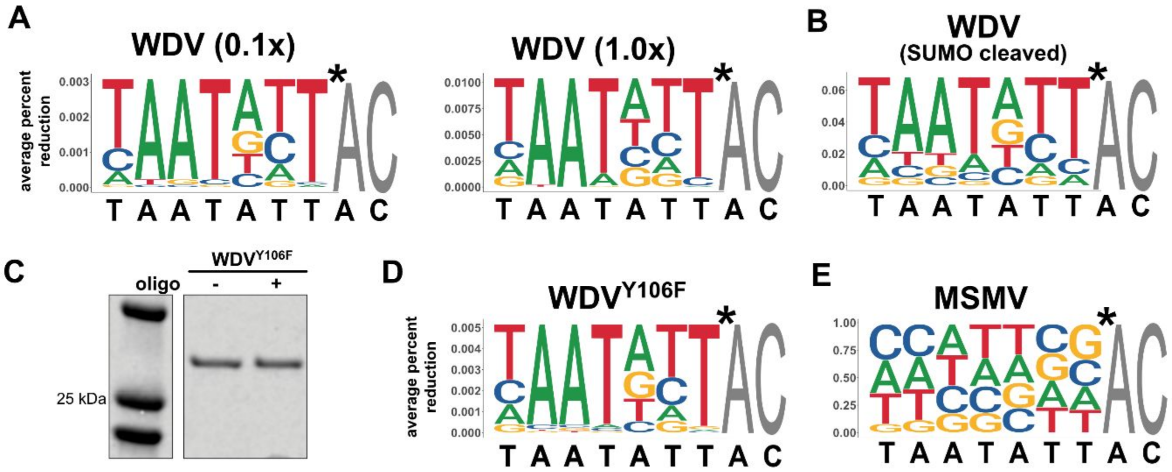

Figure S6: A, The weighted sequence logos of WDV at two lower concentrations, ten-fold less (x0.1) or equimolar (x1.0) WDV, in respect to the total 7N ssDNA library concentration. **B.** The weighted sequence logos of WDV with SUMO-cleaved. **C.** Recombinant SUMO-WDV^{Y106F} reacted with and without the 26-nt geminivirus *ori* sequence oligo under standard HUH in vitro assay conditions with 1:10 protein:oligo ratio. The SDS-PAGE gel scan is cropped to remove unrelated data. **D.** The weighted sequence logo WDV^{Y106F} and **E**. MSMV generated from HUH-seq analysis. Heights are scaled to represent the average percent reduction of each base at each position when compared to the reference library. Black sequences below each logo of the cognate nonanucleotide *ori* sequences from each respective virus.

Supplementary Table S2: Individual BASA and contact values for protein-DNA interactions in co-crystal structures

		PCV2+10-mer (6WDZ)			
		BASA	Hbond	Vdw	combine
T_{-7}	base	74.33	0	16	16
	ribose	8.61	0	1	1
	phos	0.00	0	0	0
	total	82.94	0	17	17
A_{-6}	base	24.74	3	10	13
	ribose	10.15	0	1	1
	phos	0.00	0	0	0
	total	34.89	3	11	14
G_{-5}	base	10.35	2	13	15
	ribose	11.08	1	3	4
	phos	0.00	0	0	0
	total	21.43	3	16	19
T_{-4}	base	6.20	1	3	4
	ribose	28.34	1	4	5
	phos	12.31	0	0	0
	total	46.85	2	7	9
A_{-3}	base	0.00	0	0	0
	ribose	14.71	0	7	7
	phos	23.34	2	3	5
	total	38.05	2	10	12
T_{-2}	base	0.00	0	0	0
	ribose	4.79	0	0	0
	phos	16.41	1	3	4
	total	21.20	1	3	4
T_{-1}	base	3.47	1	3	4
	ribose	32.32	2	8	10
	phos	10.51	0	0	0
	total	46.30	3	11	14
A_{+1}	base	37.85	1	17	18
	ribose	18.55	0	6	6
	phos	16.54	1	3	4
	total	72.94	2	26	28
C_{+2}	base	40.44	4	10	14
	ribose	46.97	0	7	7
	phos	2.12	0	0	0
	total	89.53	4	17	21
C_{+3}	base	28.36	0	3	3
	ribose	31.39	0	5	5
	phos	4.60	0	0	0
	total	64.35	0	8	8
	TOTAL	518.48	20	126	146

		WDV+10-mer (6WE0)			
		BASA	Hbond	Vdw	combine
T_{-7}	base	27.52	2	7	9
	ribose	7.16	0	0	0
	phos	0.00	0	0	0
	total	34.68	2	7	9
A_{-6}	base	25.21	2	10	12
	ribose	26.59	0	5	5
	phos	1.28	0	0	0
	total	53.08	2	15	17
A_{-5}	base	6.72	2	8	10
	ribose	20.40	1	9	10
	phos	7.69	0	0	0
	total	34.81	3	17	20
T_{-4}	base	2.88	0	2	2
	ribose	29.24	0	5	5
	phos	4.02	0	0	0
	total	36.14	0	7	7
A_{-3}	base	20.76	0	0	0
	ribose	0.47	0	4	4
	phos	0.00	0	0	0
	total	21.23	0	4	4
T_{-2}	base	0.00	0	0	0
	ribose	5.67	0	2	2
	phos	13.72	1	1	2
	total	19.39	1	3	4
T_{-1}	base	5.21	2	2	4
	ribose	30.17	2	5	7
	phos	12.00	0	5	5
	total	47.38	4	12	16
A_{+1}	base	21.05	0	11	11
	ribose	30.15	0	11	11
	phos	22.90	2	1	3
	total	74.10	2	23	25
C_{+2}	base	56.57	4	10	14
	ribose	35.36	0	7	7
	phos	5.82	0	0	0
	total	97.75	4	17	21
C_{+3}	base	27.20	0	3	3
	ribose	28.62	0	5	5
	phos	24.03	0	0	0
	total	79.85	0	8	8
	TOTAL	498.41	18	113	131

		WDV+8-mer (6WE1)			
		BASA	Hbond	Vdw	combine
A_{-6}	base	38.64	2	16	18
	ribose	36.64	0	6	6
	phos	0.00	0	0	0
	total	75.29	2	22	24
A_{-5}	base	7.60	2	5	7
	ribose	20.59	1	5	6
	phos	9.56	0	1	1
	total	37.75	3	11	14
T_{-4}	base	2.23	0	2	2
	ribose	29.93	0	6	6
	phos	1.71	0	0	0
	total	33.87	0	8	8
A_{-3}	base	0.00	0	0	0
	ribose	16.88	0	3	3
	phos	10.07	0	0	0
	total	26.94	0	3	3
T_{-2}	base	0.00	0	0	0
	ribose	1.82	0	0	0
	phos	23.89	1	2	3
	total	25.71	1	2	3
T_{-1}	base	5.38	0	2	2
	ribose	22.93	0	4	4
	phos	0.00	0	0	0
	total	28.31	0	6	6
A_{+1}	base	23.78	0	7	7
	ribose	27.21	1	9	10
	phos	21.08	2	2	4
	total	72.07	3	18	21
C_{+2}	base	66.18	4	10	14
	ribose	60.26	0	8	8
	phos	7.26	0	0	0
	total	133.69	4	18	22
	TOTAL	433.63	13	88	101

Table S2: Breakdown of all protein:DNA contacts and BASA values calculated by DNAproDB using default positions with ribose and phosphate interactions turned on.

Supplementary Figure S7: Discovery and validation of a target sequence exclusively cleaved by PCV2 Rep

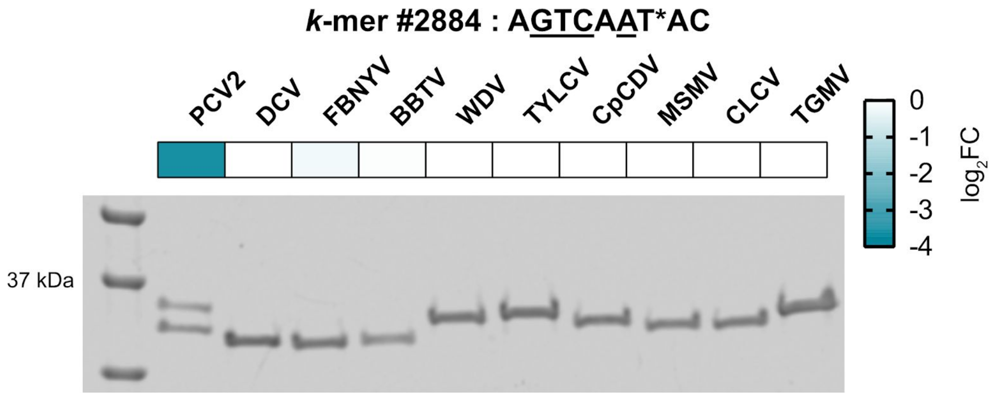

Figure S7: Single row heat map shows $\log_2$FC for each Rep treatment fromHUH-seq analysis for *k*-mer #2884, which bears a AGTCAAT sequence. There are four substitutions underlined at positions -6, -5, -4, and -2 with respect to the circovirus cognate nonanucleotide *ori* sequence. Below the heatmap is a respective SDS-PAGE gel highlighting covalent adduct formed under the standard HUH in vitro cleavage assay with a 1:2 protein:oligo ratio where only PCV2 forms adduct with *k*-mer #2884, and no detectable adduct is formed with any of the other 9 Reps.

Rep protein sequences:

>PCV2
PSKKNGRSGPQPHKRWVFTLNNPSEDERKKIRDLPISLFDYFIVGEEGNEEGRTPHLQGFANFVKKQTFN
KVKWYLGARCHIEKAKGTDQQNKEYCSKEGNLLMECGAPRSQGQR

>DCV
MAKSGNYSYKRWVFTINNPTFEDYVHVLEFCTLDNCKFAIVGEEKGANGTPHLQGFLNLRSNARAAALEE
SLGGRAWLSRARGSDEDNEEYCAKESTYLRVGEPVSKGRSS

>FBNYV
MARQVICWCFTLNNPLSPLSLHDSMKYLVYQTEQGEAGNIHFQGYIEMKKRTSLAGMKKLIPGAHFEKRR
GTQGEARAYSMKEDTRLEGPWEYGEFVP

>BBTV
MARYVVCWMFTINNPTTLPVMRDEIKYMVYQVERGQEGTRHVQGYVEMKRRSSLKQMRVFFPGAHLEK
RKGSQEEARSYCMKEDTRIEGPFEFG

>WDV
MASSSTPRFRVYSKYLFLTYPQCTLEPQYALDSLRTLLNKYEPLYIAAVRELHEDGSPHLHVLVQNKLRAS
ITNPNALNLRMDTSPFSIFHPNIQAAKDCNQVRDYITKEVDSDVNTAEWGTFVAVSTPGRKDRDAD

>TYLCV
MPRLFKIYAKNYFLTYPNCSLSKEEALSQLKKLETPTNKKYIKVCKELHENGEPHLHVLIQFEGKYQCKNQ
RFFDLVSPNRSAHFHPNIQAAKSSTDVKTYVEKDGNFIDFGVSQIDGRSARGGQQSANDAYAEAL

>CpCDV
MPSASKNFRLQSKYVFLTYPKCSSQRDDLFQFLWEKLTPFLIFFLGVASELHQDGTTHYHALLQLDKKPCI
RDPSFFDFEGNHPNIQPARNSKQVLDYISKDGDIKTRGDFRDHKVSPRKSDAR

>MSMV
MSHTSFRFRAKNVFLTYPRCPIGPEFLCDHLWNLVTPYDPLYVHVAQENHKDGGLHSHVLIQTRIEISTFD
PTYFDYTGTSIPGAVVFHPNIQACRNVRDCLAYIRKNTINEVSKGA

>CLCV
MPRNPKSFRLAARNIFLTYPQCDIPKDEALQMLQTLSWSVVKPTYIRVAREEHSDGFPHLHCLIQLSGKSN
IKDARFFDITHPRRSANFHPNIQAAKDTNAVKNYITKDGDYCESG

>TGMV
MPSHPKRFQINAKNYFLTYPQCSLSKEESLSQLQALNTPINKKFIKICRELHEDGQPHLHVLIQFEGKYCC
QNQRFFDLVSPTRSAHFHPNIQRAKSSSDVKTYIDKDGDTLVWGEFQVDGRSA

>PCV^{Y96F}
PSKKNGRSGPQPHKRWVFTLNNPSEDERKKIRDLPISLFDYFIVGEEGNEEGRTPHLQGFANFVKKQTFN
KVKWYLGARCHIEKAKGTDQQNKE**F**CSKEGNLLMECGAPRSQGQR

>WDV^{Y106F}
MASSSTPRFRVYSKYLFLTYPQCTLEPQYALDSLRTLLNKYEPLYIAAVRELHEDGSPHLHVLVQNKLRAS
ITNPNALNLRMDTSPFSIFHPNIQAAKDCNQVRD**F**ITKEVDSDVNTAEWGTFVAVSTPGRKDRDAD

>WDVc1
MASSSTPRFRVYSKYLFLTYPQCTLEPQYALDSLRTLLNKYEPLYIAAVRELHEDGSPHLHVLVQNKLRAS
ITNPNALNLRMDTSPFSIF**RCHIE**AAKDCNQVRDYITKEVDSDVNTAEWGTFVAVSTPGRKDRDAD

References

1. Chandler,M., de la Cruz,F., Dyda,F., Hickman,A.B., Moncalian,G. and Ton-Hoang,B. (2013) Breaking and joining single-stranded DNA: the HUH endonuclease superfamily. *Nat. Rev. Microbiol.*, **11**, 525–538.
